# Epidermal growth factor alters silica nanoparticle uptake and improves gold-nanoparticle-mediated gene silencing in A549 cells

**DOI:** 10.3389/fnano.2023.1220514

**Published:** 2023-07-17

**Authors:** Eva Susnik, Amelie Bazzoni, Patricia Taladriz-Blanco, Sandor Balog, Aura Maria Moreno-Echeverri, Christina Glaubitz, Beatriz Brito Oliveira, Daniela Ferreira, Pedro Viana Baptista, Alke Petri-Fink, Barbara Rothen-Rutishauser

**Affiliations:** 1 Adolphe Merkle Institute, University of Fribourg, Fribourg, Switzerland; 2 International Iberian Nanotechnology Laboratory, Braga, Portugal; 3 i4HB, UCIBIO, Departamento de Ciências da Vida, Faculdade de Ciências e Tecnologia, Universidade NOVA de Lisboa, Caparica, Portugal; 4 Department of Chemistry, University of Fribourg, Fribourg, Switzerland

**Keywords:** epidermal growth factor, nanoparticles, silica, gold, endocytosis, molecular mechanisms, gene silencing, nanomedicine

## Abstract

**Introduction:**

Delivery of therapeutic nanoparticles (NPs) to cancer cells represents a promising approach for biomedical applications. A key challenge for nanotechnology translation from the bench to the bedside is the low amount of administered NPs dose that effectively enters target cells. To improve NPs delivery, several studies proposed NPs conjugation with ligands, which specifically deliver NPs to target cells via receptor binding. One such example is epidermal growth factor (EGF), a peptide involved in cell signaling pathways that control cell division by binding to epidermal growth factor receptor (EGFR). However, very few studies assessed the influence of EGF present in the cell environment, on the cellular uptake of NPs.

**Methods:**

We tested if the stimulation of EGFR-expressing lung carcinomacells A549 with EGF affects the uptake of 59 nm and 422 nm silica (SiO_2_) NPs. Additionally, we investigated whether the uptake enhancement can be achieved with gold NPs, suitable to downregulate the expression of cancer oncogene *c-MYC*.

**Results:**

Our findings show that EGF binding to its receptor results in receptor autophosphorylation and initiate signaling pathways, leading to enhanced endocytosis of 59 nm SiO_2_ NPs, but not 422 nm SiO_2_ NPs. Additionally, we demonstrated an enhanced gold (Au) NPs endocytosis and subsequently a higher downregulation of *c-MYC*.

**Discussion:**

These findings contribute to a better understanding of NPs uptake in the presence of EGF and that is a promising approach for improved NPs delivery.

## 1 Introduction

Inorganic nanoparticles (NPs), including amorphous silica (SiO_2_) and gold (Au) NPs, are finding increasing applications in the biomedical fields, such as drug delivery (Vivero-Escoto et al., 2010; Beik et al., 2019), as carriers for nucleic acids (Roy et al., 2005; Vinhas et al., 2017), tissue regeneration (Ghosh and Webster, 2021) and biosensing (Santra et al., 2001; Slowing et al., 2007; Liu et al., 2015). Upon administration in the bloodstream, NPs encounter several physical and biological barriers, (Bourquin et al., 2018), which decrease NPs delivery efficiency (*i.e*., the dose of administered NPs that reaches the target cells) (Wilhelm et al., 2016; Dai et al., 2018). Additionally, the NPs delivery efficiency strongly depends on the NPs physicochemical characteristics, mainly, size, morphology, surface chemistry as well as cell type and NPs interaction with the complex biological environment (Rennick et al., 2021). In contrast to numerous studies comparing the effect of NPs features on their cellular uptake (Huang et al., 2010; Annika Mareike et al., 2015; de Boer et al., 2022), understanding the influence of external stimuli present in the cell microenvironment on the NPs uptake efficiency remains scarce.

A deep understanding of the mechanisms contributing to NPs internalization is the first step for efficient NPs delivery to target cells (Rennick et al., 2021). The principal route of NPs uptake into cells is known as endocytosis, and it is generally classified into phagocytosis, macropinocytosis, clathrin-mediated endocytosis (CME), caveolae-mediated endocytosis (CvME) and clathrin-/caveolae-independent endocytosis (CIE) (Zhao and Stenzel, 2018). The endocytic mechanisms may occur constitutively at the cell membrane or are triggered by cell exposure to various natural molecules, such as the epidermal growth factor (EGF) (Rennick et al., 2021). The synthesis of EGF can be induced by many cell types and is involved in the regulation of cell growth, differentiation, proliferation, migration, and endocytosis (Boonstra, 1995; Pastore et al., 2008). In fact, EGF is a well-known contributor to cancer progression (Mitsudomi and Yatabe, 2010). EGF acts by binding to epidermal growth factor receptor (EGFR), thereby causing receptor dimerization and autophosphorylation of the tyrosine residues in the cytoplasmic tail (Santos da Silva et al., 2021). EGFR autophosphorylation results in the recruitment of several adaptor and signaling proteins and the activation of mitogen-activated protein kinase (MAPK) and phosphatidylinositol 3-kinase (PI3K/AKT) downstream signaling cascades (Lemmon and Schlessinger, 2010). Important components of the PI3K/AKT signaling involve RAC1 (Rac family small GTPase 1) and CDC42 (Cell division control protein 42 homolog) proteins, which indirectly regulate endocytosis via F-actin polymerization (Kwon et al., 2000; Wennerberg and Der, 2004; Schoentaube et al., 2009; Koivusalo et al., 2010). Several studies described the involvement of EGF in the stimulation of NPs endocytosis. Nakase *et al*. observed increased uptake of 100 nm exosomes in three different cell lines (human pancreas carcinoma-derived MIA PaCa-2 cells, human pancreas adenocarcinoma-derived BxPC-3 cells, and A431 cells) in the presence of EGF. This effect has been associated with overexpression of RAS protein, actin reorganization, plasma membrane ruffling, and an increased rate of macropinocytosis (Nakase et al., 2015). Using the same cell line, Phuc *et al*. showed the stimulatory effect of EGF on the 50 nm polystyrene NPs uptake. The addition of EGF caused the aggregation of EGFRs on the cell membrane and subsequent activation of the CME pathway, thus enhancing NPs internalization together with the EGF-EGFR complex (Phuc and Taniguchi, 2017).

In this study, we shed light on the cell stimulation with EGF via activation of EGFR and RAC1, and CDC42 proteins as well as modulation of endocytic mechanisms. Lung epithelial carcinoma cells (A549) were selected as a model cancer cell line due to their high EGFR expression level and wide use in NPs-cell interaction studies (Dos Santos et al., 2011; Mohamed et al., 2011; Nowak et al., 2014; Annika Mareike et al., 2015). We hypothesized that A549 cell stimulation with EGF could modulate the cellular uptake of NPs. The uptake of 59 nm and 422 nm SiO_2_ NPs in A549 cells was assessed in the absence and presence of EGF under different co-exposure conditions. The internalization of small particles is believed to occur via CME, CvME and macropinocytosis, while larger NPs are predominantly internalized via macropinocytosis (Schmid and Conner, 2003; Park and Oh, 2014). Next, we tested whether EGF stimulation can modulate the uptake of Au NPs, coated with polyethylene glycol (PEG) to ensure NPs stability (Au@PEG) and carrying antisense oligonucleotides (ASOs), complementary to *c-MYC* transcript (Au@PEG@c-myc) (Conde et al., 2013b). The motivation to investigate AuNPs alongside SiO_2_ NPs stemmed from the use of SiO_2_ NPs as a model system to determine the impact of NPs size on EGF-mediated effects. Given the greater complexity of AuNPs and their widespread use as gene therapy vectors, we were intrigued to investigate whether EGF could stimulate their endocytosis as well, thereby improving the therapeutic efficacy. The active transport of AuNPs occurs mostly through endocytosis via pathways like CME, CvME, CIE, and macropinocytosis (Brandenberger et al., 2010; He et al., 2017) and is influenced by properties such as size, surface charge, and polarity (Conde et al., 2013a; Fernandes and Baptista, 2017; Roma-Rodrigues et al., 2020). We aimed to target the *c-MYC* gene, as it is a known transcription factor involved in cell cycle homeostasis (Hermeking et al., 2000; García-Gutiérrez, Delgado, and León, 2019). In cancer cells, this gene is frequently deregulated and thus a promising therapeutic target. ASOs can specifically silence the *c-MYC* expression by using antisense single-stranded DNA complementary to the target sequence in the cell (Conde et al., 2012). By cell stimulation with EGF, we aim to enhance the uptake of Au@PEG@c-myc NPs and subsequently achieve a higher *c-MYC* silencing effect.

Our findings emphasize the importance of investigating NPs uptake in the presence of EGF within the cellular environment and considering diverse NPs properties and co-exposure strategies, which influence NPs endocytosis. By elucidating the interplay between EGF and NPs uptake, this study contributes to the advancement of targeted therapeutic strategies and the development of more effective treatments for cancer.

## 2 Materials and methods

### 2.1 Cell cultures

Human alveolar epithelial type II cells (A549) were obtained from the American Tissue Type Culture Collection (ATCC^®^ CRM-CCL-185^™^, Rockville, MD, United States). Cells were cultured in RPMI 1640 (Gibco, Luzern, Switzerland) completed with heat-inactivated 10 vol% foetal bovine serum (FBS), 2 mM L-Glutamine (100 Units/mL, Gibco, Luzern, Switzerland) and penicillin/streptomycin (100 μg/mL, Gibco, Luzern, Switzerland)(specified as cRPMI) and kept at 37°C, 95% relative humidity and 5% pCO_2_. Cell splitting was performed at 80%–90% confluence twice per week using a mixture of 0.05 vol% Trypsin-ethylenediaminetetraacetic acid (EDTA) (Gibco, Luzern, Switzerland). A549 cells were seeded at a density of 52.000 cells/cm^2^ in a 6-well plate (Corning, Reinach, Switzerland), 8-well Ibidi μ-Slide chambers (Cat. No. 80827, Ibidi, Graefelfing, Germany), 24-well or 96-well plates (SPL Life Sciences, Cat. 13485, Gyeonggi-do, Korea), depending on the experimental technique used. Cells were grown for 24 h at 37°C, 95% relative humidity, and 5% pCO_2_.

### 2.2 Receptor expression

Cultivation conditions for A549-EGFR-GFP, ARPE-19, HeLa, Calu-3, Caco-2, HT-29, and THP-1 cell lines are included in Supplementary Table S1. All cell lines were cultivated for 24 h at 37°C, 95% relative humidity, and 5% pCO_2_ and then further processed for Western blot and confocal laser scanning microscopy (refer to the corresponding sections).

### 2.3 Cell stimulation with epidermal growth factor

After the seeding for 24 h, A549 cells were serum starved for 24 h at 37°C, 5% CO_2_, and 95% humidity. Serum starvation is a widely employed technique in experiments involving growth factors and has been used for synchronizing cells by arresting them in the G0/G1 phase of the cell cycle (Kawamura et al., 2004; Bryant et al., 2007; Kim et al., 2016). To ensure result comparability across different conditions and time points, serum starvation was consistently employed in all tested conditions, including control groups. Human recombinant EGF protein (Cat. PHG-0313, Thermo Fisher Scientific, Zug, Switzerland) was prepared in a cRPMI in final concentration 100 ng/mL and added to cells at different time points (5 min, 10 min, 15 min, 30 min, 1 h, 4 h, and 24 h). During this time, cells were incubated at 37°C, 5% CO_2_, and 95% humidity. After, cells were lysed and the protein contents were analyzed for EGFR and RAC1/CDC42 expression via Western blot. Additionally, Rac1 expression was quantified via RT-qPCR.

### 2.4 Cell viability

#### 2.4.1 Lactate dehydrogenase assay

Levels of the enzyme lactate dehydrogenase (LDH) in the supernatants after different exposures were measured in triplicates according to the manufacturer’s protocol (Roche Applied Science, Mannheim, Germany). Application of 0.2 vol% Triton X-100, diluted in the cell culture medium for 24 h served as a positive control for membrane rupture, while untreated cells served as a negative control. The absorbance of the colorimetric product Formazan was determined spectrophotometrically (Benchmark microplate reader, BioRad, Cressier, Switzerland) at 490 nm, with a reference wavelength of 630 nm. LDH values are presented as fold increase values relative to untreated cells.

#### 2.4.2 MTS assay

After exposures, cells were washed with PBS and then 100 μL of MTS reagent (1:5 v/v in cRPMI; CellTiter 96 AQueous One Solution Cell Proliferation Assay, Cat. 63581, Promega, Southampton, United Kingdom) was added to each well. Untreated cells were considered as a positive control and cells exposed to 50% inhibitory concentration (IC_50_) of doxorubicin-hydrochloride (15 μM; Cat. D2975000, Merck, New Jersey, US) for 24 h as a negative control. The plates were incubated at 37°C, 5% CO_2_in a humid environment for 30 min. The absorbance of solubilized formazan was recorded at 490 nm using a microplate reader (Infinite M200, Tecan, Männedorf, Switzerland). Averaged blank values were subtracted from sample values. The corrected values were presented as percentages relative to the untreated cells.

### 2.5 Western blot

Treated cells were lysed in M-PER buffer (M-PER^™^ Tissue Protein Extraction Reagent, Cat. 78501, Thermo Fisher Scientific, Zug, Switzerland) containing EDTA-free Halt^™^ Protease Inhibitor Cocktail (Cat. 78425, Thermo Fisher Scientific, Zug, Switzerland) and sodium fluoride (Cat. 27860, 20 mM, VWR, Dietikon, Switzerland). The amount of protein in the lysates was quantified using Nanodrop^™^ 2000 spectrophotometer (Thermo Fisher Scientific, Zug, Switzerland). Samples were mixed with Laemmli loading buffer, heated at 95°C for 10 min, and then loaded onto 7.5% polyacrylamide gels (20 μg/lane). A molecular weight marker mPAGE^®^ Color Protein Standard (Cat. MPSTD4, Sigma-Aldrich, Buchs, Switzerland) was used to identify the bands on a gel. Gels were transferred to polyvinylidene difluoride (PVDF) membranes (Bio-Rad, Hercules, CA, United States) at 80–100 V for 1 h. The transfer of proteins to the membrane was confirmed using 0.1 w/v.% solution of Ponceau-S stain (Cat. 141194, Sigma-Aldrich, Buchs, Switzerland) and 0.05 vol% acetic acid in Milli-Q water. The membrane was blocked in 5 vol% BSA, prepared in wash buffer (20 mM Tris-HCl, pH 7.6, 150 mM NaCl, and 0.1 vol% Tween-20) for 1 h at room temperature. Detection of the protein of interest was achieved by probing the membrane with the following primary antibodies: Anti-EGFR (1 μg/mL, Cat. AF231, R&D systems, Abingdon, United Kingdom), anti-Phospho-EGFR Tyr1068 antibody (1:1,000, Cat. D7A5 XP, Cell Signaling Technology, Allschwil, Switzerland), anti-Rac1/Cdc42 (1:1,000, Cat. 4,651, Cell Signaling Technology, Allschwil, Switzerland), anti-Phospho-Rac1/Cdc42 S71 (1:1,000, Cat. 2,461, Cell Signaling Technology, Allschwil, Switzerland), anti-GAPDH (1 μg/mL, sc-47724, Santa Cruz Biotechnology, Heidelberg, Germany) and anti-α-tubulin (1 μg/mL, Cat. sc-5286, Santa Cruz Biotechnology, Heidelberg, Germany). Membranes were incubated with primary antibodies overnight at 4°C on a shaker. Bound primary antibodies were visualized with secondary IgG antibodies conjugated to horseradish peroxidase: anti-goat (Cat. HAF017, R&D, Abingdon, United Kingdom) at 1:1,000 (EGFR), anti-rabbit (Cat. HAF008, R&D, Abingdon, United Kingdom) at 1:2000 (Rac1/Cdc42) and anti-mouse (Cat. HAF007, R&D, Abingdon, United Kingdom) at 1: 4,000 (α-tubulin and GAPDH). All antibodies were diluted in a blocking buffer. Washing with TBST (0.1 vol% Tween-20 in TBS) was performed between each step. HRP-conjugated secondary antibodies (KPL, Gaithersburg, MD) were visualized using the chemiluminescent HRP detection reagent Immobilon Forte Western HRP substrate (Cat. WBLUF0020, Sigma-Aldrich, Buchs, Switzerland) under chemiluminescent detection system (ImageQuant^™^ LAS4000, Chicago, US). Changes in protein levels were quantified by densitometry using ImageJ software. The relative values of the samples were determined by giving an arbitrary value of 1.0 to the respective control samples of each experiment.

### 2.6 Real-time quantitative PCR

#### 2.6.1 RAC1 expression

Cells treated with EGF for different time points were washed three times with ice-cold PBS (pH 7.4, Cat. 10010023, Thermo Fisher Scientific, Zug, Switzerland) followed by the addition of 200 μL lysis buffer per well. Lysis buffer was prepared by mixing BL buffer and 1-thioglycerol (ReliaPrep^™^ RNA Miniprep Systems, Promega, Madison, WI, United States) in a 1:100 (*v/v*) ratio. Total RNA was extracted from the cell lysates using the ReliaPrep^™^ RNA Miniprep Systems according to the manufacturer’s recommendations (Promega, Madison, WI, United States). RNA yield and quality were determined using a Nanodrop 2000 spectrophotometer (Thermo Fisher Scientific, Zug, Switzerland). Only RNA samples with an optical density ratio of 260/280 ≥ 1.8 were considered for further experiments. Complementary DNA (cDNA) was synthesized using a mixture of Omniscript RT system (Cat. 205113, Qiagen, Hilden, Germany), Oligo dT primers (Microsynth, Balgach, Switzerland), and RNasin ribonuclease inhibitor (Cat. NZ2611, Promega, Madison, WI, United States) and incubated for 1 h at 37°C in thermal cycler (Labcycler Gradient, SensoQuest, Göttingen, Germany). Real-Time PCR amplification was performed using the 7,500 fast real-time PCR system(Applied Biosystems, Thermo Fisher Scientific, Waltham, MA, United States) by mixing 2 μL of 9-fold diluted cDNA with 5 μL of SYBR-green master mix (Fast SYBR Green master mix, Applied Biosystems, Thermo Fisher Scientific, Waltham, MA, United States), 2 μL of nuclease-free water (Promega, Madison, WI, United States), and 2 μL of primer mix (91 nM). Data were analyzed by the comparative threshold cycle (Ct) method (2^-ΔΔCt^) (Livak and Schmittgen, 2001), where relative gene expression is given by quantification of the gene of interest (*RAC1*) relative to internal standard genes (glyceraldehyde-3-phosphate dehydrogenase - *GAPDH* and tyrosine 3-monooxygenase/tryptophan 5-monooxygenase activation protein zeta - *YWHAZ*), normalized to the control. Primers were purchased from Thermo Fisher Scientific (Zug, Switzerland). Detailed information on primer sequence and cycling conditions can be found in Supplementary Table S2.

#### 2.6.2 c-MYC expression

A549 cells were exposed to Au@30%PEG NPs and Au@30% PEG@c-myc NPs and total RNA was extracted using RNA Purification Kit (Cat. Z3100, Promega, Madison, United States) according to the manufacturer's specifications. Only RNA samples with an optical density ratio 260/280 ≥ 1.8 were used for further experiments. RNA yield and quality were determined using Nanodrop spectrophotometer (ND-1000, NanoDrop Technologies, Wilmington, United States). RT-qPCR amplification was performed via One-step NZY RT-qPCR Green kit (Cat. MB343, NZYTech, Lisbon, Portugal) in a Qiagen Rotor-Gene Q cycler (Qiagen, Hilden, Germany). A 10 μL reaction mix was prepared to contain 1 μL RNA(10 ng/μL), 5 μL One-step NZY qPCR Green master mix (2x), 0.4 μM c-MYC or 0.6 μM 18s (housekeeping) primers (STABVida, Caparica, Portugal), 0.4 μL reverse transcriptase and diethyl pyrocarbonate-treated water (DEPC). At least three independent repeats for each experiment were carried out. Relative expression levels were calculated based on the 2^-ΔΔCT^ method (Livak and Schmittgen, 2001). The sequences of primers and the cycling steps are shown in Supplementary Table S3.

### 2.7 Cell exposures to endocytic markers

A549 cells were seeded on 6-well plates (52.000 cells/cm^2^) and grown for 24 h in a complete medium (10% FBS) at 37°C, 5% pCO_2_, and 95% humidity. Cells were serum starved for an additional 24 h. Before cell exposure, the following endocytic markers were prepared in cRPMI: 70 kDa dextran-FITC (250 μg/mL, Cat. 46945, Sigma-Aldrich, Buchs, Switzerland), Transferrin-Alexa Fluor488 (10 μg/mL, Cat. 009-540-050, Jackson Immunoresearch, Cambridgeshire, United Kingdom) and cholera toxin subunit B (CTxB; 100 ng/mL; Cat. C34778, Thermo Fisher Scientific, Zug, Switzerland). Cells were exposed to markers and 100 ng/mL EGF simultaneously for 1 h, 4 h, and 24 h. As a control, the uptake of endocytic markers was traced in cells in the absence of EGF. Markers’ internalization and uptake have been determined by confocal microscopy and flow cytometry.

### 2.8 Nanoparticle synthesis and characterization

Fluorescently labelled SiO_2_-BDP FL NPs of 59 nm and 422 nm were synthesized by following an adaptation of the Stöber method (Stober et al., 1968) as described in previous studies (Susnik et al., 2020; A; Lee et al., 2022).

Au@citrate NPs were synthesized via the citrate reduction method (Lee and Meisel, 1982). The concentration of added HAuCl_4_ was 1 mM. Initially, 12 nm Au@citrate NPs were incubated for 16 h with 0.01 mg/mL of a thiolated polyethylene glycol (PEG; M_w_: 356 Da, Cat. 672572, Sigma-Aldrich, Buchs, Switzerland), to achieve 100% saturation of the NP’s surface (Au@100%PEG NPs). Excess PEG was removed by centrifugation at 14.000 *g* for 30 min (three times), and the level of PEG coverage on the Au NPs surface was evaluated from the UV-Vis absorbance spectra of PEGylated AuNPs at 412 nm and quantification of free thiol-PEG in the supernatant via the Ellman’s assay (Cat. D8130, Sigma-Aldrich, Buchs, Switzerland) (Hassan et al., 2010; Baptista et al., 2013; McCully et al., 2018). For silencing experiments, Au@PEG NPs with 30% saturation of the NPs’ surface (Au@30%PEG NPs) were prepared (0.003 mg/mL PEG) and subsequently functionalized with the thiolated stem-looped antisense oligonucleotides (ASO) complementary to the *c-MYC* transcript (5′-GCGCCCATTTCTTCCAGATATCCTCGCTGGGCGC-3′) (Au@PEG@c-Myc NPs) as previously described (Rosa et al., 2012; Conde et al., 2013a). Following ASO quantification via UV/Vis spectroscopy (UV mini-1240 spectrophotometer, Shimadzu, Duisburg, Germany), each ASO was added to the Au@30%PEG NPs in a 1:150 (AuNPs: ASO) ratio. The number of ASOs bonded to the Au NPs’ surface was determined via NanoDrop spectrophotometer by subtracting the number of ASOs present in the supernatants recovered from the NPs washes from the initial amount of ASOs incubated with NPs. For control experiments, Au@30%PEG NPs without ASOs were prepared.

Both, SiO_2_-BDP FL NPs and Au@PEG NPs core size and morphology were examined via Tecnai Spirit transmission electron microscope (TEM; FEI Technai G2 Spirit, Thermo Fisher Scientific, Waltham, MA, United States) equipped with a Veleta CDD camera (Veleta, Olympus, Tokyo, Japan). The core diameters and size distributions were calculated from TEM images using ImageJ software (National Institutes of Health, Bethesda, MD, United States). NPs hydrodynamic diameter, polydispersity index, and ζ-potential were characterized in Milli-Q water and complete cell culture media (cRPMI) by a Malvern Zetasizer Nano ZS at 37°C, scattering angle 173° and laser wavelength 633 nm (Malvern, Zetasizer Nano series, United Kingdom). Each measurement was repeated 5 times and then the average and standard deviation were calculated. Additional characterization of Au@PEG NPs in cRPMI was performed by a DLS spectrometer LS Instruments AG (Fribourg, Switzerland) at the scattering angle of 90° and laser wavelength 660 nm. The auto-correlation functions for each NPs dispersed have been analyzed and represented as fit (+residuals) corresponding to the estimated distribution of the intensity-weighted hydrodynamic radius.

### 2.9 Cell exposure to nanoparticles

A549 cells were seeded at a density 52.000 cells/cm^2^, grown for 24 h in cRPMI. Serum starvation was conducted 24 h prior to the introduction of NPs. Subsequently, the NPs were mixed with a serum-containing medium before being applied to the cells. This approach allowed us to investigate the specific effects of the NPs on the cells while maintaining a serum-containing environment that supports NPs stability and provides essential factors for sustaining cell viability. All NPs were taken from the stock in Milli-Q water and freshly dispersed in cRPMI immediately before the exposure. Cells were exposed to 2 mL/well of 59 nm and 422 nm SiO_2_-Bodipy NPs previously dispersed in cRPMI at a final concentration of 50 μg/mL. In terms of NPs concentrations, this equals 1.7 × 10^11^ of 59 nm NPs/mL and 5.6 × 10^8^ of 422 nm NPs/mL. For experiments using 8-well μ-Slide Ibidi chambers (Ibidi, Graefelfing, Germany), cells were treated with 50 μg/mL NPs previously suspended in cRPMI. Five different exposure experiments were performed.

- Single exposure: NPs were pre-mixed in cRPMI before cell exposure for 4 h or 24 h;

**Figure:**
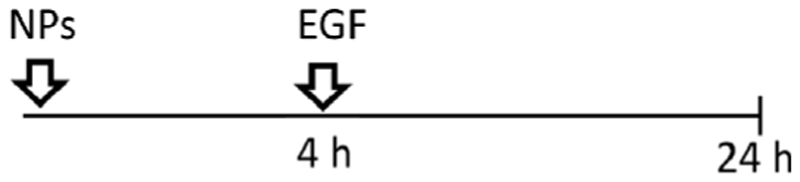
- Simultaneous co-exposure: 100 ng/mL of EGF and NPs were pre-mixed in cRPMI before cell exposure for 4 h or 24 h;

**Figure:**
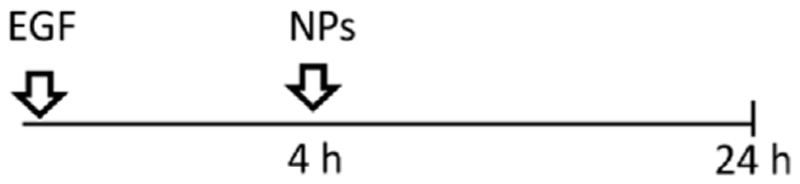
- Sequential co-exposure:
  (1) NPs were applied first to the cells for 4 h, followed by the addition of EGF for 20 h (NPs were not removed between steps);

**Figure:**
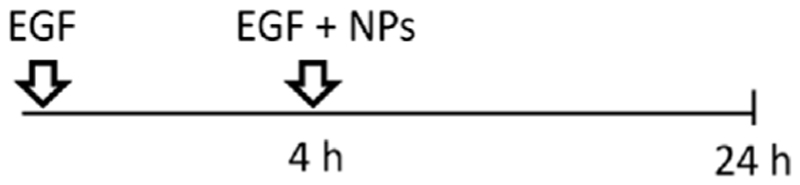
  (2) EGF was applied first to the cells for 4 h, followed by the addition of NPs for 20 h (EGF was not removed between steps);

**Figure:**
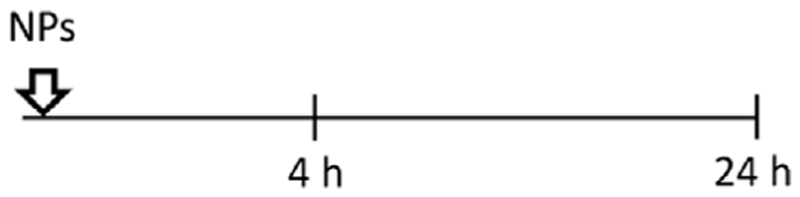
  (3) EGF was applied first to the cells for 4 h, then EGF was removed and replaced by a pre-mixed suspension of EGF and NPs for 20 h.

**Figure:**
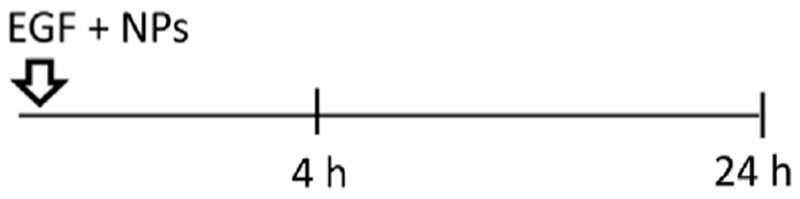

Cells, exposed to SiO_2_-BDP FL NPs were harvested for flow cytometry analysis or underwent fixation and staining procedures for confocal microscopy imaging (refer to the corresponding sections).

To determine the uptake of Au NPs in A549 cells, 52.000 cells/cm^2^ were seeded in 24-well plates and treated with 0.5 mL/well of Au@100%PEG NPs, freshly dispersed in cRPMI at a final concentration of 10 nM (1.1 × 10^14^ NPs/mL). For gene silencing, the same volume of Au@30%PEG NPs or Au@30%PEG@c-myc NPs was added at a final concentration of 0.54 nM (6.1 × 10^12^ NPs/mL). Single, simultaneous, and sequential co-exposure condition (2) were used. Cells were prepared for ICP-AES analysis and RT-qPCR (refer to the corresponding sections). The exposures were performed in three biological replicates. Under all conditions, cells were kept in the incubator at 37°C, 5% CO_2_, and 95% humidity.

### 2.10 Flow cytometry

After cell exposures to SiO_2_-Bodipy NPs, cells grown in 6-well cell culture plates were washed three times with PBS, detached using 0.05% Trypsin-EDTA (200 μL), and incubated for 5 min at 37°C. To stop the trypsin activity, 600 μL of cRPMI was added. Cell suspensions from wells with the same treatment were combined in 5 mL polystyrene FACS tubes (Cat. 352054, Corning, Reinach, Switzerland). After, cells were centrifuged for 5 min at 300 × *g* and the cell pellet was resuspended in cold flow cytometry buffer, containing 1% bovine serum albumin (BSA) and 1 mM EDTA (Sigma-Aldrich, Buchs, Switzerland), dissolved in PBS (w/v). To distinguish between live and dead cells, 1 μg/mL of 4', 6-diamidino-2-phenylindole (DAPI; Cat. D9542, Sigma-Aldrich, Buchs, Switzerland) was prepared in flow cytometry buffer (v/v) and incubated with cells for 10 min at 4°C. Data acquisition was performed on a BD LSR FORTESSA (BD Biosciences, San Jose, CA, United States) equipped with a violet laser (Excitation: 405 nm; DAPI) and blue laser (Excitation: 488 nm; Dextran-FITC and Transferrin-AF488). The emission bandpass filters 450/50 (DAPI) and 530/30 (Dextran-FITC and Transferrin-AF488) were used. Flow cytometry data for 20.000 recorded events were analyzed using the FlowJo software (version 10.8.1, TreeStar, Woodburn, OR, United States).

### 2.11 Inductively coupled plasma atomic emission spectroscopy

The internalization of the Au@100%PEG NPs and Au@30% PEG@c-myc NPs in A549 cells was evaluated using the inductive coupled plasma atomic emission spectroscopy technique (ICP-AES, Horiba Jobin-Yvon, Ultima, Palaiseau, France) equipped with a 40.68 MHz RF generator, Czerny-Turner monochromator with 1 m focal length, auto-sampler AS500, and CMA (Concomitant Metals Analyser). After treatments, cells were washed with PBS and trypsinized with 150 μL TrypLE TM Express (Cat. 12604-021, Gibco, New York, United States) for 5 min at 37°C. The detached cells were counted via Trypan blue dye exclusion test using a Neubauer chamber (Cat. 631-0926, VWR, Dietikon, Switzerland) and then centrifuged at 500 *g* for 5 min. The supernatant was removed and the cell pellet was stored at -20 °C. One day before the analysis, 1 mL of freshly prepared aqua regia (HCl: HNO_3_ = 3:1) was added to the samples. A standard curve of gold was recorded to quantify the amount of intracellular gold. The mass of gold obtained from the analysis was normalized to the final cell number.

### 2.12 Confocal microscopy

Cells were washed three times with PBS and fixed in 4% paraformaldehyde (Cat.158127, Sigma-Aldrich, Buchs, Switzerland), dissolved in PBS (v/v) for 15 min. Unspecific antibody binding was blocked with cell incubation in 1 vol% bovine serum albumin (Cat. A9418, Sigma-Aldrich, Buchs, Switzerland), dissolved in PBS for 1 h. To test EGFR expression, cells were stained with primary goat anti-human EGFR antibody (1 μg/mL, Cat. AF231, R&D systems, Abingdon, United Kingdom), diluted in blocking buffer, and incubated for 1 h. Afterward, donkey anti-goat Alexa 647-conjugated secondary antibody (1:500, ab150131, Abcam, United Kingdom) was added for 1 h. For experiments involving the uptake of endocytic markers and NPs, cells were additionally stained with a rhodamine-phalloidin probe (0.66 μM in 1% BSA in PBS (w/v), Cat. R415, Invitrogen, Thermo Fisher Scientific, Zug, Switzerland) for 1 h. Cell nuclei were counterstained using 4,6-diamidino-2-phenylindole (DAPI; 1 μg/mL, Sigma Aldrich, Buchs, Switzerland) in PBS (v/v) for 10 min. Samples were washed three times with PBS between each step and all experiments were performed at room temperature. As a final step, cells were kept in 300 μL PBS in Ibidi chambers at 4°C until imaging. The images were acquired using Leica STELLARIS SP5 confocal laser scanning microscope equipped with hybrid detectors (HyD) and an HC PL APO CS2 63x/1. 40 OIL objective (Leica Microsystems, Germany). Excitation was set to 405 nm (DAPI), 488 nm (SiO_2_-Bodipy NPs, Dextran-FITC, and Transferrin-AF488), 561 nm (rhodamine-phalloidin), and 638 nm (EGFR and CTxB). The emission was acquired from 410 to 502 nm (DAPI), 502-556 nm (SiO_2_-Bodipy NPs, Dextran-FITC, and Transferrin-AF488), 566–643 nm (rhodamine-phalloidin), and 643–755 nm (EGF and CTxB). Images were analyzed using Fiji software.

### 2.13 Dark-field microscopy

Cells were seeded into round glass coverslips (6 × 10^4^ cells/slide; surface area: 1.13 cm^2^) and supplemented with 0.5 mL of cRPMI. Cells were grown overnight, serum-starved, and exposed to Au@100%PEG NPs alone (single) or with EGF (simultaneously and sequentially). Next, the cells were washed three times with PBS to remove non-interacting NPs. The cell monolayer was fixed with 4% paraformaldehyde (PFA; Cat. P6148, Sigma-Aldrich, Buchs, Switzerland) in PBS (v/v), followed by mounting onto glass slides with glycerol (Cat. G5516, Sigma-Aldrich, Buchs, Switzerland). The samples were visualized using a 50× objective lens with a numerical aperture of 0.50 in a Cytoviva enhanced darkfield microscopy (Cytoviva Inc., Auburn, AL, United States). Images were analyzed using Fiji software.

### 2.14 Statistical analysis

Statistical analysis was performed using GraphPad Prism 8 software (GraphPad Software Inc., San Diego, CA, United States). One-way analysis of variance (ANOVA) with Dunnett’s or Tukey’s multiple comparisons test or unpaired Student’s t-test was used to compare values among the different treatments. Statistically significant values among the treatments are explained in the figure captions.

## 3 Results and discussion

### 3.1 Expression of epidermal growth factor receptor

Different human cell lines (lungs: A549 and Calu-3; colon: HT-29 and Caco-2; cervix: HeLa; retina: ARPE-19 and macrophages: THP-1) were screened to determine EGFR expression levels using a Western blot (Supplementary Figure S1). The highest EGFR expression was detected in A549, ARPE-19, and HeLa cells, compared to HT-29, Calu-3, and Caco-2 cells. On the other hand, no EGFR expression was observed in THP-1 macrophages. The observed differences in EGFR expression among cell lines can be attributed to the variable phenotypes, physiological function, and stages of cellular differentiation (Real et al., 1986; Kriegs et al., 2019). Alveolar epithelial cell line A549 was chosen for further experiments, based on high EGFR expression and multiple studies describing this cell line as a physiologically relevant model to study the interactions between NPs and human cells (Dos Santos et al., 2011; Mohamed et al., 2011; Nowak et al., 2014; Annika Mareike et al., 2015).

### 3.2 EGF stimulates its receptor in a time-dependent manner

**Figure 1:**
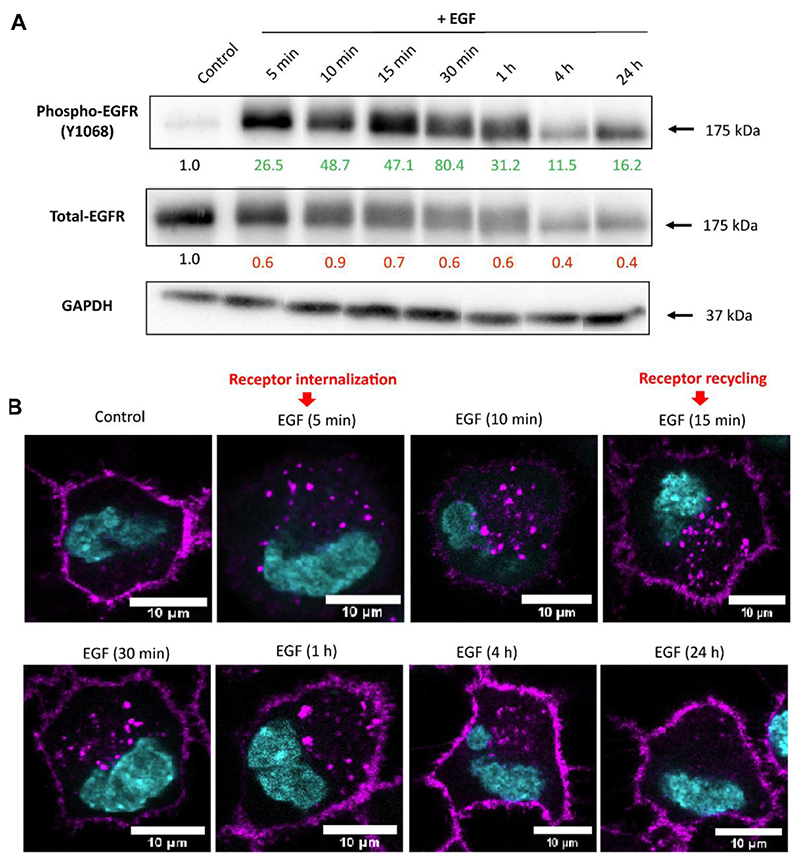
The EGFR expression upon cell stimulation with EGF. **(A)** Representative Western blot images showing expression of phosphorylated- and total-EGFR (Phospho-EGFR and Total-EGFR), upon EGF stimulation at different time points. GAPDH served as an internal control for protein loading. The average expression values of the indicated protein were determined via densitometry (Fiji software) from three independent experiments and presented as a fold change over the negative controls. Increased expression is marked in green and decreased in red. Full Western blot images including the corresponding controls for each time point and weight marker are shown in Supplementary Figure S2. **(B)** Localization of EGFR upon cell stimulation with EGF assessed via confocal microscopy. Receptor internalization was observed 5 min after A549 stimulation with EGF, followed by receptor recycling back to the membrane staring at 15 min. Magenta: EGFR;Cyan: nuclei. Scale bar: 10 μm.

We aimed to investigate whether EGF binding activates EGFR and downstream signaling pathways involved in endocytosis regulation in A549 cells. After seeding, cells were subjected to serum starvation by culturing in cRPMI without FBS for 24 h. Serum starvation was performed to synchronize cells to the same cell cycle phase, increase the sensitivity of cells to exogenously added growth factors, and reduce experimental variability. Cells were treated with recombinant human EGF in a concentration of 100 ng/mL. As previously reported, EGF enhances cellular uptake of NPs in a dose-dependent manner and 100 ng/mL of EGF showed higher enhancement than lower concentration of EGF (Phuc and Taniguchi, 2017). First, we tested whether the cell stimulation with EGF changes the expression of total and phosphorylated (Tyr1068) EGFR in a time-dependent manner (Figure 1A). Western blot analysis shows an initial increase in EGFR phosphorylation with the peak (~80-fold increase) at 30 min upon EGF stimulation, as supported by other studies (Lahusen et al., 2007; Pinilla-Macua et al., 2017). However, at later time points the levels of phosphorylated-EGFR gradually decreased, but remained phosphorylated at all-time points in the presence of EGF. In contrast, we noted a decrease in the expression of total EGFR upon EGF stimulation over time, which could be related to receptor degradation (Capuani et al., 2015; Yao et al., 2020). EGFR protein levels are expected to decrease when stimulated with a high concentration of EGF due to the presence of a negative feedback mechanism that regulates downstream signaling events (Tanaka et al., 2018). As a result, the EGFR protein is targeted for degradation, leading to a reduction in its overall abundance. To gain a deeper understanding of the EGFR intracellular paths, A549 cells in which the genomic EGFR gene has been endogenously tagged with a green fluorescent protein gene (A549-EGFR-GFP) have been used to follow receptor localization upon EGF treatment. Using confocal microscopy, we observed that EGF stimulation causes the initial internalization of EGFR from the membrane to the cytoplasm, followed by EGFR recycling back to the cell surface (Figure 1B). Internalized EGFR can thus follow different pathways. One possibility is the partial degradation of EGFR within lysosomes, which helps to maintain cellular homeostasis. Additionally, a fraction of internalized EGFR can be recycled back to the plasma membrane, thereby maintaining the abundance of EGFR for sustained signalling. Furthermore, *de novo* synthesis of EGFR can also occur, contributing to the pool of available EGFR for subsequent activation by EGF. We propose those mechanisms to be crucial for the sustained phosphorylation of EGFR, despite a decrease in total-EGFR fraction. In agreement with our study, (Chi et al., 2011), described that continuous recycling of the EGFR to the cell surface might sustain signaling.

### 3.3 The effect of EGF on RAC1/CDC42 protein expression

**Figure 2:**
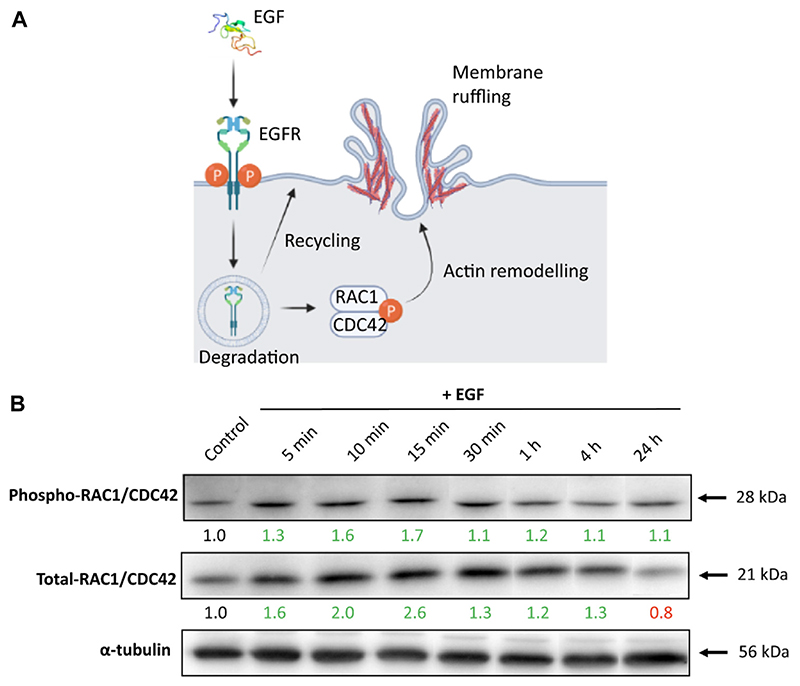
The RAC1/CDC42 expression upon cell stimulation with EGF. **(A)** Schematic depicting EGFR activation (phosphorylation) by EGF and activation of downstream effectors RAC1/CDC42, which subsequently enhance actin remodeling, and membrane ruffling/endocytosis. Illustration created with BioRender.com (2023), agreement number JO24WALJCR. **(B)** A549 cells stimulated with EGF-induced Phospho-RAC1/CDC42 and Total-RAC1/CDC42 protein expression levels in a time-dependent manner. α-tubulin served as an internal control for protein loading. The average expression values of the indicated protein were determined via densitometry from five independent experiments and presented as a fold change over the negative controls. Increased expression is marked in green and decreased in red. Full Western blot images including the corresponding controls for each time point and weight marker are shown in Supplementary Figure S4.

EGF binding causes EGFR dimerization and subsequent autophosphorylation of its tyrosine residues, which triggers multiple signal transduction pathways, including members of the Rho family of GTPases RAC1 and CDC42 (Kurokawa et al., 2004; Law et al., 2014). Phosphorylation of RAC1 and CDC42 enables their binding with downstream effectors, required for regulation of endocytosis via F-actin polymerization (Figure 2A) (Dharmawardhane et al., 2000; Lemmon and Schlessinger, 2010; Pinilla-Macua et al., 2017). Our goal was to follow temporal changes in RAC1/CDC42 phosphorylation levels in response to EGF stimulation. We hypothesized that EGF stimulation could enhance endocytosis through the activation of CDC42 and RAC1. Protein levels of phosphorylated and total RAC1/CDC42 at different time points have been determined via Western blot and the *RAC1* gene expression via RT-qPCR. The most notable increase in the level of phosphorylated (~1.7 fold) and total (~2.6-fold) RAC1/CDC42 levels upon EGF treatment was noted within the first 15 min, indicating time-dependent regulation of RAC1/CDC42 proteins in response to EGF stimulation. At later time points, we observed that RAC1/CDC42 remained phosphorylated, albeit to a lesser extent. The sustained phosphorylation of EGFR and the continuous presence of EGF could potentially contribute to the sustained phosphorylation of RAC1/CDC42 (Figure 2B). In contrast, there were no significant differences in the *RAC1* gene expression upon cell stimulation with EGF (Supplementary Figure S3). We suggest that pathway regulation in response to EGF does not result in transcriptional changes in the RAC1 gene, but rather occurs at the protein level through post-translational modifications. Of note, although interference of EGF with the RAC1/CDC42 effectors is observed, additional signaling pathways or phosphorylation sites of RAC1/CDC42 are likely to be involved at the onset of the EGF stimulus (Dise et al., 2007; Takenawa and Shiro, 2007).

### 3.4 EGF induces the uptake of endocytic markers

**Figure 3:**
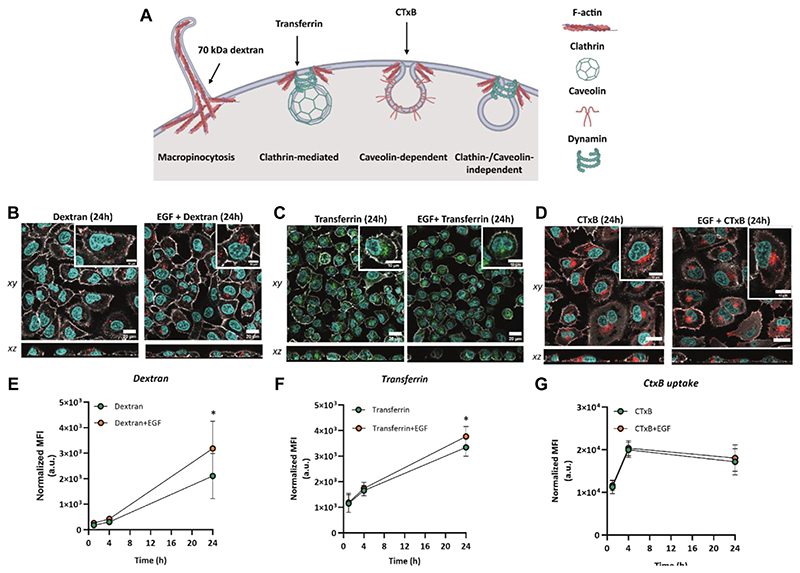
Cellular uptake of endocytic markers 70 kDa dextran, transferrin and cholera toxin subunit **(B)**. **(A)** Endocytic pathways for the uptake of 70 kDa dextran (macropinocytosis marker) and transferrin (clathrin-mediated endocytosis marker). Illustration created with BioRender.com (2023), agreement numberAW24WALG9E. Confocal laser scanning micrographs showing the uptake of **(B)** 70 kDa dextran (red), **(C)** transferrin (green) and **(D)** cholera toxin subunit B (CTxB) in the absence and presence of EGF after 24 h of simultaneous exposure. Grey: F-actin, Cyan: nuclei. Scale bar: 20 μm. Zoom-in images of single cells are shown in the insets. Scale bar: 10 μm. Flow cytometry assessment of **(E)** 70 kDa dextran (250 μg/mL) uptake, **(F)** transferrin (10 μg/mL) uptake and **(G)** CTxB (100 ng/mL) uptake without EGF or added simultaneously with EGF. Graphs represent the median fluorescence intensity (MFI), with subtracted background values from untreated cells. Data are presented as mean ± standard deviation (n = 3). Statistically significant differences between the two groups were assessed via paired *t*-test: *p ≤ 0.05.

To evaluate the effect of EGF stimulation on endocytic pathway activation, A549 cells were exposed simultaneously to EGF and dextran, transferrin or cholera toxin subunit B (CTxB) for 1 h, 4 h, and 24 h. Fluorescently labelled 70 kDa dextran has been employed to monitor uptake via macropinocytosis (Li et al., 2015), transferrin was selected to test activation of clathrin-mediated endocytosis (Rennick et al., 2021), whereas CTxB is a marker for caveolin-dependent endocytosis (Pelkmans and Helenius, 2002) (Figure 3A). Based on LDH activity, there was no significant cytotoxicity in cells exposed to markers in the absence or presence of EGF at all time points (Supplementary Figure S5A). Confocal microscopy data revealed internalization of 70 kDa dextran, transferrin and CTxB in the presence and absence of EGF (Figures 3B–D). The markers were found intracellularly within the first hour upon exposure and the fluorescent signal, corresponding to the endocytic marker uptake, increased over time in the absence or presence of EGF (Supplementary Figure S6). Based on the flow cytometry results, we observed a modest, albeit statistically insignificant increase in the uptake of both dextran and transferrin at 4 h of EGF stimulation. At 24 h after the exposure, the uptake of 70 kDa dextran in the presence of EGF increased ~1.5-fold, whereas the uptake of transferrin increased ~1.1-fold compared to their uptake in unstimulated cells (Figures 3D,E). This indicates an enhanced activation of macropinocytosis and CME in the presence of EGF. Since the highest effect of EGF stimulation was observed on the uptake of 70 kDa dextran, this indicates a greater enhancement of macropinocytosis. On the other hand, EGF did not induce a notable alteration in the uptake of CTxB. These results suggest that while EGF might be involved in the upregulation of macropinocytosis and caveolin-mediated endocytosis, its impact on caveolae-dependent endocytosis appears to be negligible. Our observation is in agreement with the previous studies, showing the involvement of EGF in modulating mainly macropinocytosis and CME (Nakase et al., 2015; Phuc and Taniguchi, 2017).

### 3.5 Nanoparticle characterization

According to the United States Food and Drug Administration definition, which defines materials up to one micron as nanomaterials, all size ranges used in this article were considered as NPs (Boverhof et al., 2015).

**Figure 4:**
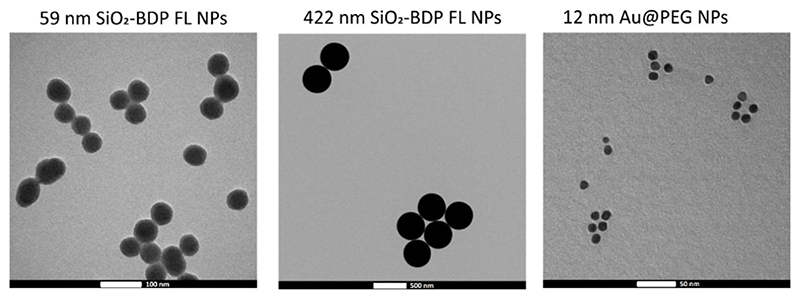
Representative TEM micrographs of 59 nm SiO_2_-BDP FL NPs, 422 nm SiO_2_-BDP FL NPs, and 12 nm Au@100%PEG NPs.

Amorphous SiO_2_ NPs of two different sizes were synthesized and labelled with Bodipy fluorescein dye (BDP FL) to investigate their cellular uptake in the presence of EGF. As determined from the transmission electron microscopy (TEM) images the average core diameters of SiO_2_-BDP FL NPs were 59 ± 6 nm and 422 ± 14 nm (Figure 4). Table 1 summarizes the hydrodynamic diameter, polydispersity index (PDI), and ζ-potential, measured via dynamic light scattering (DLS) in Milli-Q water and cRPMI at 24 h of incubation at 37°C. The hydrodynamic diameters of NPs dispersed in Milli-Q water were 71 ± 1 nm and 484 ± 9 nm, whereas in cRPMI we detected 91 ± 8 nm and 458 ± 41 nm. In the presence of EGF, the hydrodynamic diameters of NPs dispersed in Milli-Q water were 71 ± 1 nm and 469 ± 5 nm, whereas in cRPMI diameters corresponded to 90 ± 5 nm and 464 ± 40 nm (Supplementary Figure S7). The presence of EGF did not affect protein corona formation on the NPs surface. Core and hydrodynamic size distributions of SiO_2_-BDP FL NPs are represented in Supplementary Figure S7. SiO_2_-BDP FL NPs, with large negative ζ-potentials (>-30 mV), indicate that NPs are colloidally stable. The polydispersity index (PDI) of both NPs sizes was less than 0.1, thus implying narrow size distribution (Mudalige et al., 2019).

Au@citrate NPs with a mean core diameter of 12 ± 1 nm (Figure 4) were synthesized and functionalized with poly (ethylene glycol) (PEG) spacers to increase their stability (Conde et al., 2012). Au NPs with 100% PEG saturation of the NP’s surface (Au@100%PEG NPs) were used for uptake studies. To conduct silencing experiments, we prepared Au@PEG nanoparticles (NPs) with a 30% saturation of the NPs’ surface and functionalized them with thiolated stem-looped antisense oligonucleotides (ASOs) complementary to the *c-MYC* transcript (Au@30%PEG NPs@c-myc). 30% PEGylated AuNPs without ASOs (Au@30%PEG NPs) served as a negative control for silencing. Mean hydrodynamic diameters of Au@PEG NPs in water corresponded to 23 ± 1 nm for Au@100%PEG NPs, 24 ± 2 nm for Au@30%PEG NPs, and 24 ± 1 nm for Au@30%PEG@c-myc NPs. In cRPMI, mean hydrodynamic diameters varied based on the NPs surface coatings: 22 ± 1 nm for Au@100%PEG NPs, 40 ± 10 nm for Au@30%PEG NPs and 44 ± 4 nm for Au@30%PEG@c-myc NPs (Table 1). Au@PEG NPs had negative surface charges and PDI values above 0.2. The differences in values between NPs dispersed in Milli-Q water and cRPMI possibly arise due to the presence of proteins, salts, and macromolecules in cRPMI that can influence the measurement process as well as affect the NPs stability when adsorbed on the surface (i.e., protein corona) (Pino et al., 2014; Moore et al., 2015; Ke et al., 2017; Fong et al., 2019). The additional TEM micrographs, core- and hydrodynamic size distributions as well as auto-correlation function of Au@100%PEG NPs, Au@30%PEG NPs, and Au@30%PEG@c-myc NPs are included in Supplementary Figure S8A,B,C includes absorbance spectra for Au@30%PEG NPs and Au@100%PEG NPs and a standard calibration curve for PEG to evaluate the surface coverage with PEG.

### 3.6 Cellular uptake of silica NPs upon EGF stimulation

**Figure 5:**
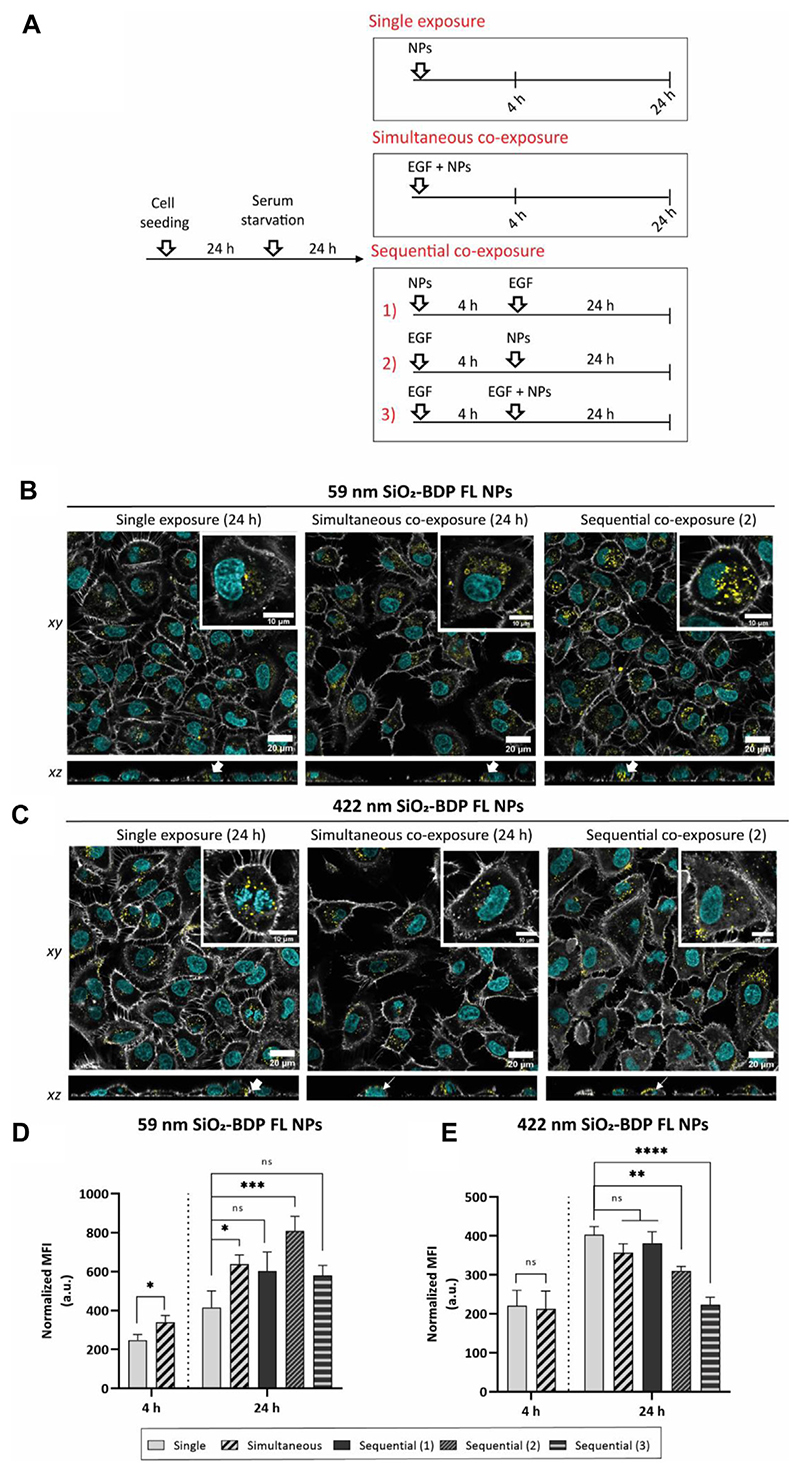
Uptake of 59 nm and 422 nm SiO_2_-BDP FL NPs upon cell stimulation with EGF. **(A)** Schematic representation of different experimental co-exposure scenarios. Confocal laser scanning microscopy data showing the association of **(B)** 59 nm SiO_2_-BDP FL NPs and **(C)** 422 nm SiO_2_-BDP FL NPs with A549 cells under single exposure, simultaneous co-exposure, and sequential co-exposure condition (2) with EGF. Single and co-exposure conditions are represented schematically in the Materials and methods. For clarity, representative images of internalized NPs under single, simultaneous, and sequential co-exposure condition (2) at 24 h are shown; the remaining images are included in Supplementary Figure S9. Thick and thin white arrows on z-projections indicate intracellular and surface-bound NPs, respectively. Yellow: NPs, Grey: F-actin, Cyan: nuclei. Scale bar: 20 μm. Representative single cells are shown in insets; scale bar: 10 μm.The uptake of **(D)** 59 nm SiO_2_-BDP FL NPs and **(E)** 422 nm SiO_2_-BDP FL NPs in A549 cells has been assessed via flow cytometry and presented as median fluorescence intensity of BDP FL signal (MFI in arbitrary units), with subtracted background values from untreated cells. Statistically significant differences among the groups (n = 3) were assessed via paired *t*-test (4 h time-point) or One-way ANOVA with Dunnett’s test for multiple comparisons (24 h time-point): *p ≤ 0.05; **p ≤ 0.01; ***p ≤ 0.001; ****p ≤ 0.0001; ns—not significant.

Having demonstrated that two different endocytic pathways are induced upon EGF stimulation, cells were exposed to 59 nm SiO_2_-BDP FL NPs and 422 nm SiO_2_-BDP FL NPs in the absence and presence of EGF. Internalization of smaller particles occurs via various endocytic mechanisms, including CME and macropinocytosis, while larger NPs are primarily internalized through macropinocytosis (Schmid and Conner, 2003; Park and Oh, 2014; Sousa de Almeida et al., 2021). Experiments were performed by following single exposure or simultaneous and sequential co-exposure conditions as described in Materials and methods and Figure 5A. Incubation periods of 4 h and 24 h were analyzed to differentiate between short- and long-term cell exposure effects.

Confocal microscopy revealed the uptake of both NPs with cells at 4 h upon exposure and an increase in intracellular NPs accumulation at 24 h (Supplementary Figure S9). We noted an increase in intracellular fluorescence of 59 nm SiO_2_-BDP FL NPs under simultaneous and sequential co-exposure condition 2) with EGF, compared to a single NPs exposure (Figure 5B). There was no visual difference observed between single NPs exposure and sequential co-exposure conditions 1) or 3) with EGF (Supplementary Figure S9B). On the other hand, a significant number of 422 nm SiO_2_-BDP FL NPs can be seen in contact with the outer cell membrane but are not internalized (Figure 5C). Cells internalized the lowest amount of 422 nm SiO_2_-BDP FL NPs under sequential co-exposure condition 3), as compared to other conditions (Supplementary Figure S9C).

To obtain semi-quantitative data on the intracellular deposition of SiO_2_-BDP FL NPs, flow cytometry analysis has been performed. The uptake of 59 nm SiO_2_-BDP FL NPs increased in the presence of EGF at 4 h and 24 h (Figure 5D). The highest NPs uptake was achieved under sequential co-exposure condition 2) (~2-fold increase compared to single NPs exposure), followed by simultaneous co-exposure with EGF (~1.5-fold increase). We hypothesize that cell prestimulation with EGF under sequential co-exposure condition 2) accelerated macropinocytosis and CME during 4 h of incubation (refer to the uptake of dextran and transferrin) and thus resulted in the highest effect on NPs uptake. However, under other sequential co-exposure approaches, no changes in NPs uptake were evidenced. If NPs were applied 4 hours before EGF (sequential co-exposure 1)), they might interfere with the EGF receptor, causing reduced EGFR sensitivity to EGF (Sousa de Almeida et al., 2023). A possible explanation for the absence of differences in NPs uptake under sequential co-exposure condition 3) can be an overstimulation of EGFR and associated pathways with the second EGF dose. This mechanism acts as a negative-feedback loop to prevent overstimulation of the EGFR axis (Haryuni et al., 2020).

Of particular interest is the EGF effect on the reduced uptake of 422 nm SiO_2_-BDP FL NPs, under sequential co-exposures 2) and 3), resulting in a ~1.3-fold and ~1.8-fold reduction of NPs uptake, respectively (Figure 5E). Given the small degree of differences, no significant changes were considered when NPs were added simultaneously or under sequential co-exposure condition 1). Confocal microscopy further demonstrated differences in the association of 422 nm SiO_2_-BDP FL NPs with cells. In the presence of EGF, some NPs were not internalized but rather adhered to the cell membrane. These observations allow us to reconcile the results from flow cytometry showing a substantial cellular internalization of NPs during single exposure and a lower uptake after co-exposure with EGF. Two different mechanisms could explain this outcome: one considers that CME became a dominant pathway over macropinocytosis in the presence of EGF (Phuc and Taniguchi, 2017). CME vesicles are cargo size limited (<200 nm), thus not able to accommodate 422 nm SiO_2_-BDP FL NPs (Canton and Battaglia, 2012). This mechanism is not very likely, since multiple studies demonstrated induced macropinocytosis upon EGF stimulation (Bryant et al., 2007; Nakase et al., 2015; Rennick et al., 2021). A second, and in our view more likely alternative, is the involvement of actin disassembly, required to complete the internalization of larger NPs. Schlam *et al*. showed evidence of F-actin breakdown and RAC1/CDC42 inactivation necessary for the termination of endocytosis of larger NPs (Schlam et al., 2015). As actin polymerizes in response to EGF stimulation, one or more components of the assembly process may become depleted over time. Replenishment of components needed for the uptake of large NPs would only occur following their disassembly or recycling. Remarkably, this mechanism was not evident during the endocytosis of smaller NPs.

As assessed via LDH assay, we did not detect any increase in cell membrane rupture as a measure of cytotoxicity in any of the NPs exposure conditions (Supplementary Figure S5B).

### 3.7 Cellular uptake and *c-MYC* silencing efficiency of gold NPs upon EGF stimulation

**Figure 6:**
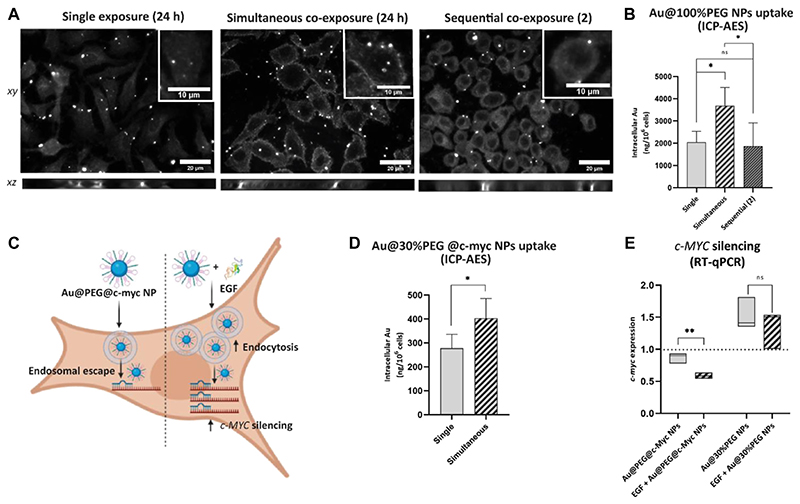
Uptake and c-MYC silencing effect of different PEGylated Au NPs upon cell stimulation with EGF. **(A)** Enhanced dark-field microscopy images (including z-stack projections) of A549 cells after 24 h exposure to Au@100%PEG NPs under single exposure (left), simultaneous co-exposure with EGF (middle) or sequential co-exposure with EGF (right). Scale bar: 20 μm. Representative single cells are shown in insets; scale bar: 10 μm. **(B)** ICP-AES measurements reveal an increased uptake of Au@100%PEG NPs under simultaneous co-exposure with EGF. Statistically significant differences among the groups (n = 5) were assessed via One-way ANOVA with Tukey’s test for multiple comparisons: *p ≤ 0.05;ns—not significant. **(C)** Scheme representing a proposed mechanism of Au@PEG@c-myc NPs for delivery of ASOs complementary to c-MYC transcript into cells. In the absence of EGF, NPs internalize by cells via endocytosis, followed by their endosomal escape in the cytoplasm. The c-MYC hairpin on NPs opens in the presence of the complementary c-MYC sequence and silences its expression. The presence of EGF increases NPs uptake, which consequently targets more c-MYC sequences, resulting in a higher silencing effect. Illustration created with BioRender.com (2023), agreement number MH24WALB85. **(D)** ICP-AES measurements reveal an increased uptake of Au@30%PEG@c-myc NPs under simultaneous co-exposure with EGF. Statistically significant differences among the groups (n = 3) were assessed via paired *t*-test: *p ≤ 0.05. **(E)** Cell exposure to Au@30%PEG@c-myc NPs simultaneously with EGF decreased c-MYC expression as confirmed via RT-qPCR. Au@30%PEG NPs served as a control to test the effect of NPs alone on c-MYC expression. Values are normalized to the untreated cells, with the expression of 1. Statistically significant differences among the groups (n = 3) were assessed via unpaired *t*-test (RT-qPCR): **p ≤ 0.01;ns—not significant.

Further studies were conducted to investigate whether the stimulation of A549 cells with EGF enhances the uptake of Au NPs and potentially improves their gene silencing potential. Using the Au@100%PEG NPs allows us better estimation of uptake due to their high stability in complete medium, while the Au@30% PEG enables the antisense oligonucleotide to bind to the “empty space” on the 30% PEG surface coverage. Experiments were performed by following single exposure set-up, or simultaneous and sequential co-exposure 2) conditions as described in Materials and methods. We used enhanced dark-field imaging to locate Au@100%PEG NPs in A549 cells and visually observed cell association with Au@100%PEG NPs under all exposure conditions (Figure 6A). The images illustrate that applied NPs form clusters that either attach to the cell membrane or are already found intracellularly (*xz*-projections). Based on raw integrated densities (i.e., the sum of pixel values) of the cells exposed to Au@100%PEG NPs obtained from dark-field images, a significant increase was observed under simultaneous NPs co-exposure with EGF as compared to single NPs exposure (Supplementary Figure S10A). ICP-AES was further used to quantify the differences in intracellular Au mass. Cells that were simultaneously exposed to the EGF and Au@100%PEG NPs internalized ~1.8-fold more NPs compared to a single NPs exposure. On the other hand, no significant changes in NPs uptake were seen, when EGF was applied 4 h before the cell exposure to Au@100%PEG NPs under sequential co-exposure condition 2) (Figure 6B).

PEGylated Au NPs functionalized with antisense oligonucleotide complementary to the *c-MYC* transcript (Au@30%PEG@c-myc NPs) were used to further investigate NPs’ effect on *c-MYC* silencing. Cells were exposed to single Au@30% PEG@c-myc NPs or simultaneously with EGF for 24 h. We focused on simultaneous exposure NPs and EGF due to the confirmed enhanced effect of EGF on Au@100%PEG NPs uptake under this condition. Figure 6C represents a proposed mechanism of Au@30% PEG@c-myc NPs for delivery of gene silencing NPs into cells in the presence or absence of EGF. Cells internalize NPs via endocytosis, followed by their endosomal escape in the cytoplasm. The *c-MYC* hairpin on NPs opens in the presence of the complementary *c-MYC* sequence in the cytoplasm and silences *c-MYC* mRNA expression. The presence of EGF increases NPs uptake, which consequently targets more *c-MYC* mRNA transcripts, resulting in a higher silencing effect. We conducted ICP-AES experiments using Au@30%PEG@c-myc NPs both under single- and simultaneous exposure with EGF, as these conditions were employed for the silencing experiments (Figure 6D). Our results confirmed ~1.5-fold enhanced uptake of Au@30%PEG@c-myc NPs when administered simultaneously with EGF. Based on RT-qPCR data to evaluate *c-MYC* expression, we observed an insignificant decrease in *c-MYC* expression after cell exposure to the Au@30%PEG@c-myc NPs alone. Simultaneous cell exposure with EGF resulted in a ~3-fold decrease of *c-MYC* expression, compared to untreated cells (Figure 6E). We propose that cell stimulation with EGF enhanced NPs uptake, which in turn contributed to a stronger *c-MYC* silencing effect. As a control, Au@30%PEG NPs without *c-MYC* silencing moiety were tested and were found to induce *c-MYC* gene expression. The upregulation of the *c-MYC* gene in response to Au@30%PEG NPs, without the silencing ASOs, may be attributed to the impact of these NPs on the expression of genes related to cell metabolism, cell-cycle progression, and division. Previous *in vitro* studies have highlighted the effects of bare and PEG-modified AuNPs on the cell cycle regulation, apoptosis, DNA repair, and metabolic stress (Pan et al., 2007; Butterworth et al., 2010; Kang et al., 2010; Chuang et al., 2013; Uz et al., 2016). It is important to note that the slight overexpression observed for the Au@30%PEG NPs is effectively counteracted by the silencing effect exerted by the ASO. By comparing the influence of Au@30%PEG NPs on *c-MYC* expression in the presence and absence of EGF, we observed no significant differences. Hence, we conclude that although Au@30% PEG NPs alone may have an impact on *c-MYC* expression, those results do not alter our conclusion regarding the role of EGF in *c-MYC* silencing.

Additionally, we investigated the effect of A549 cell treatment with Au@100%PEG NPs, Au@30%PEG NPs, and Au@PEG@c-myc in the presence and absence of EGF on cell proliferation/viability. None of the exposure conditions affected the cell proliferation/viability as assessed via MTS assay (Supplementary Figure S10B).

## 4 Conclusion

Natural molecules present in the cell environment, such as epidermal growth factor (EGF) can specifically regulate endocytosis in epithelial cells. This study describes the effect of EGF on cell signaling, the modulation of endocytic activity, and the uptake of different SiO_2_ NPs and PEGylated Au NPs in A549 lung epithelial carcinoma cells. The primary objective is to conduct a comprehensive assessment of how EGF influences cell signalling pathways and endocytosis, as this is important to better understand the uptake of NPs. SiO_2_ NPs of two different sizes were tested as a model NPs to determine the impact of NPs size on EGF-mediated NPs uptake. Considering the application of Au NPs as gene therapy vectors, we found it interesting to explore the possibility of EGF to stimulate the uptake of AuNPs and consequently enhancing therapeutic efficacy. Our findings demonstrated, that EGF exerts its biological effects by binding to its specific receptor, epidermal growth factor receptor (EGFR), and induces several downstream signaling pathways, involving RAC1/CDC42 effectors in a timedependent manner. Cell stimulation with EGF facilitated the uptake of 70 kDa dextran, a marker for macropinocytosis as well as transferrin, a marker for CME. Since EGF did not induce a notable alteration in the uptake of CTxB its impact on caveolae-dependent endocytosis appears to be negligible. Our results further highlight the importance of the internalization process of differentsized SiO_2_-BDP FL NPs in the presence of EGF. We found that cell stimulation with EGF facilitated the uptake of 59 nm SiO_2_-BDP FL NPs, in particular under simultaneous and sequential co-exposure scenario 2). In contrast, reduced uptake of 422 nm SiO_2_-BDP FL NPs was noted. These observations can be associated with the differences in F-actin dynamics during endocytosis of NPs with different sizes. Further, we investigated the potential of using EGF to enhance the cellular uptake of Au NPs coated with polyethylene glycol (PEG) and carrying antisense oligonucleotides (ASO), complementary to the *c-MYC* transcript (Au@PEG@c-myc) in A549 cells. The proposed application of such NPs would be to effectively silence the frequently upregulated *c-MYC* oncogene without side effects or toxicity, resulting in a reduced tumor size. We were able to demonstrate that cell stimulation with EGF increases the uptake of Au@PEG NPs and improves the *c-MYC* silencing efficacy of Au@PEG@c-myc NPs. In this study, we assessed the impact of EGF using SiO_2_ NPs of sizes 59 nm and 422 nm, as well as Au@PEG NPs. By investigating multiple types of NPs, we aimed to consider the diverse characteristics displayed by different NPs, including variations in size, material, and surface properties. The conclusions derived from the NPs uptake study were based on the specific NPs investigated in this study. However, conducting additional experiments with different NPs formulations would further enhance our understanding of the interplay between EGF and NPs, ultimately contributing to the development of more effective applications for NPs. In the field of nanomedicine, it is important to explore the potential of combining therapy with bioactive molecules, as this approach can increase the cellular NPs uptake and improve NPs delivery in target cells. Additionally, for new developed therapeutic NPs, simplicity and efficiency should be prioritized to facilitate their approval process.

## Supplementary Material

The Supplementary Material for this article can be found online at: https://www.frontiersin.org/articles/10.3389/fnano.2023.1220514/full#supplementary-material

Supplementary material

## Figures and Tables

**Table 1:** Physicochemical characteristics of different NPs measured via TEM and DLS.

	d_c_ ± SD (nm)	d_h_ ± SD (nm)		PDI	ζ-potential ±SD (mV)
		*H_2_0*	*cRPMI*		
SiO_2_-BDP FL NPs	59 ±6	71 ± 1	91 ± 8	0.07	- 32 ± 2
	422 ± 14	484 ± 9	458 ± 41	0.07	- 47 ± 2
Au@100%PEG NPs	12 ± 1	23 ± 1	22 ± 1	0.28	- 82 ± 7
Au@30%PEG NPs	12 ± 1	24± 2	40 ± 10	0.46	- 56± 3
Au@30%PEG@c-myc NPs	12 ± 1	24 ± 1	44 ± 4	0.32	- 64 ± 1

Abbreviations: d_c_: core diameter (TEM), d_h_: hydrodynamic diameter (DLS), PDI: polydispersity index, ζ-(zeta) potential (measured at pH 7.0), SD: standard deviation.

## Data Availability

The original contributions presented in the study are included in the article/Supplementary Materials, further inquiries can be directed to the corresponding author.
