# Supplementary material for: Epidermal growth factor alters silica nanoparticle uptake and improves gold-nanoparticle-mediated gene silencing in A549 cells
Source: Front Nanotechnol. Author manuscript; Available in PMC 2023 Nov 12. (PMC7615298; doi:10.3389/fnano.2023.1220514)
Supplement: Supplementary material [file EMS187668-supplement-Supplementary_material.docx]

Supplementary Material

Epidermal growth factor alters silica nanoparticle uptake and improves gold-nanoparticle mediated gene silencing in A549 cells


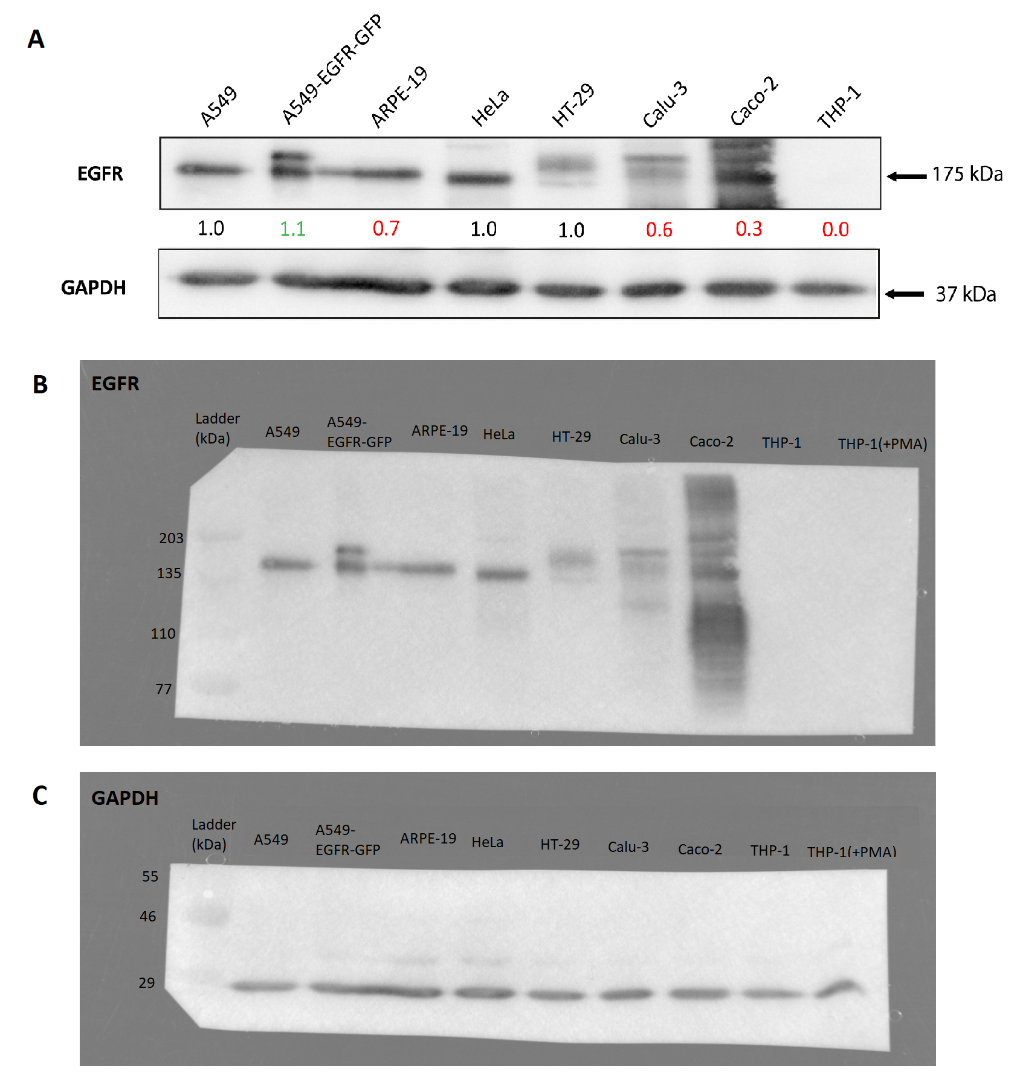


**Supplementary Figure 1.** **Expression of EGFR in different human cell lines, determined by Western blot.** **(A)** The average EGFR expression values were determined via densitometry (Fiji software) from three independent experiments and normalized to the A549 cell line. Increase in EGFR expression is marked in green and decrease in red. A549-EGFR-GFP is a cell line with EGFR gene tagged with GFP, producing two bands; lower for wild-type EGFR and upper for EGFR-GFP chimeric protein. Full western blot images with the corresponding weight marker lane showing expression of **(B)** EGFR (175 kDa), and **(C)** GAPDH (37 kDa). After the transfer, the membranes were cut in order to perform the immunoblotting for different proteins of interest in the same membrane. This can be visualized by the corresponding ladder (mPAGE® Color Protein Standard, Cat. #MPSTD4, Sigma-Aldrich) according to the band size (kDa).


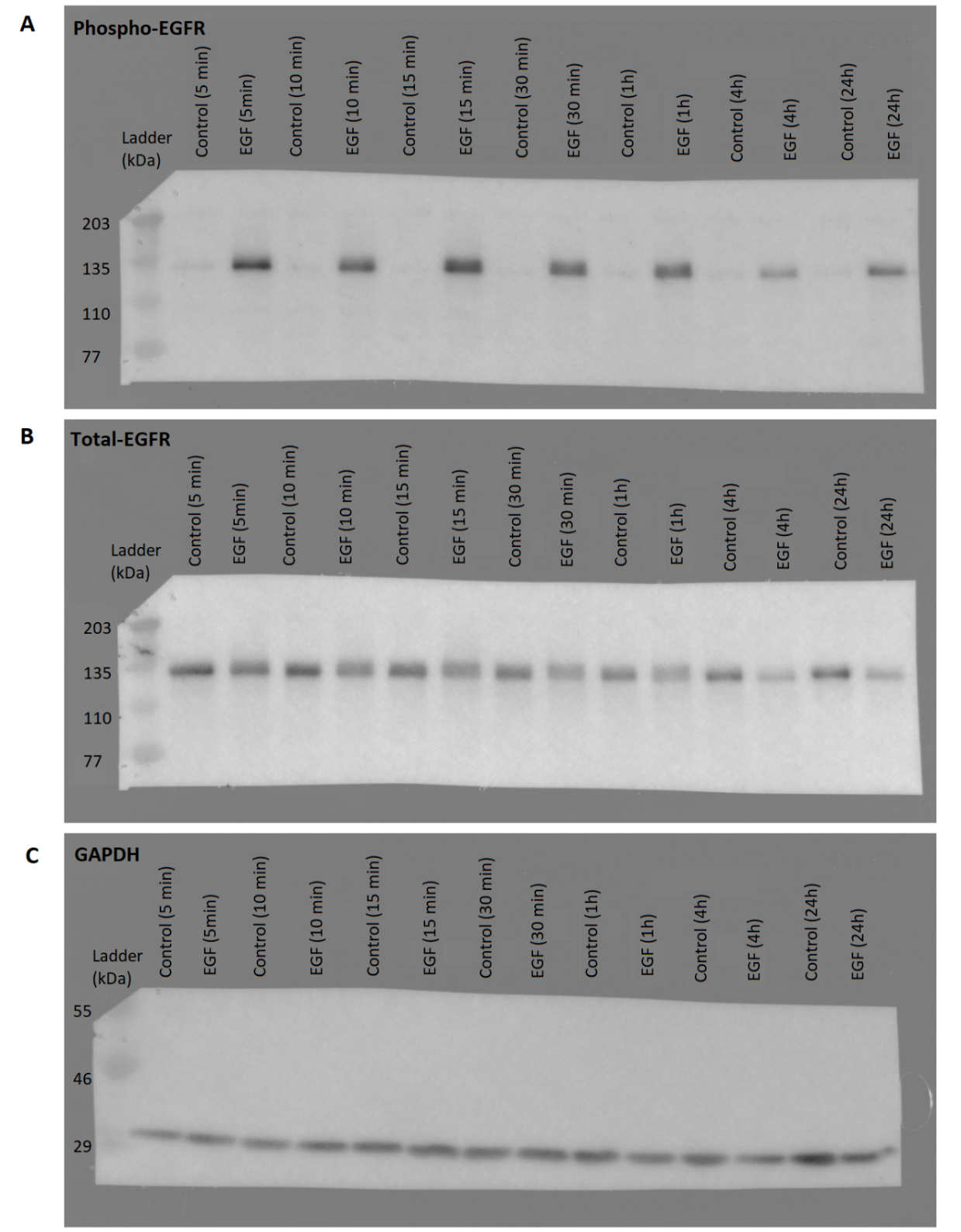


**Supplementary Figure 2.** **Full western blot images with the corresponding weight marker lane showing EGFR expression.** Western blot images showing expression of **(A)** phosphorylated-EGFR (175 kDa), **(B)** total-EGFR (175 kDa), and **(C)** GAPDH (37 kDa), upon EGF stimulation at different time points. GAPDH served as an internal control for protein loading. For each time point, the corresponding controls (i.e., cells without EGF treatment) are shown next to the EGF-treated samples. After the transfer, the membranes were cut in order to perform the immunoblotting for different proteins of interest in the same membrane. This can be visualized by the corresponding ladder (mPAGE® Color Protein Standard, Cat. #MPSTD4, Sigma-Aldrich) according to the band size (kDa).


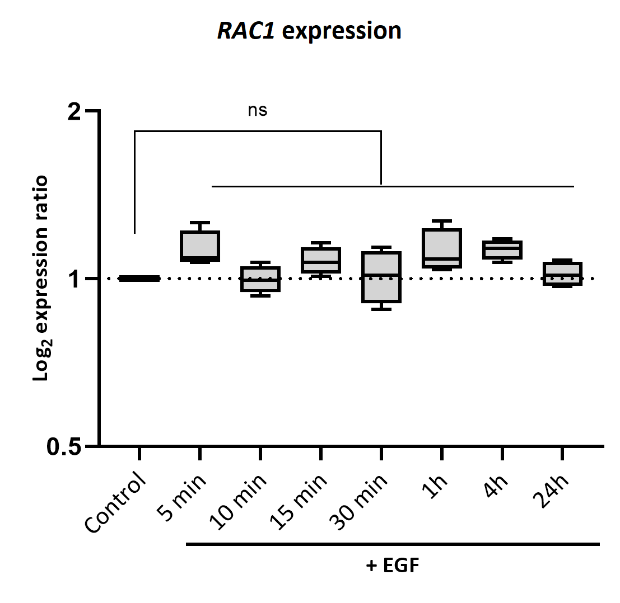


**Supplementary Figure 3. *RAC1* expression upon cell stimulation with EGF.** Real-time PCR data obtained from four independent experiments represent the expression of the RAC1 gene upon stimulation with EGF at different time points.

**
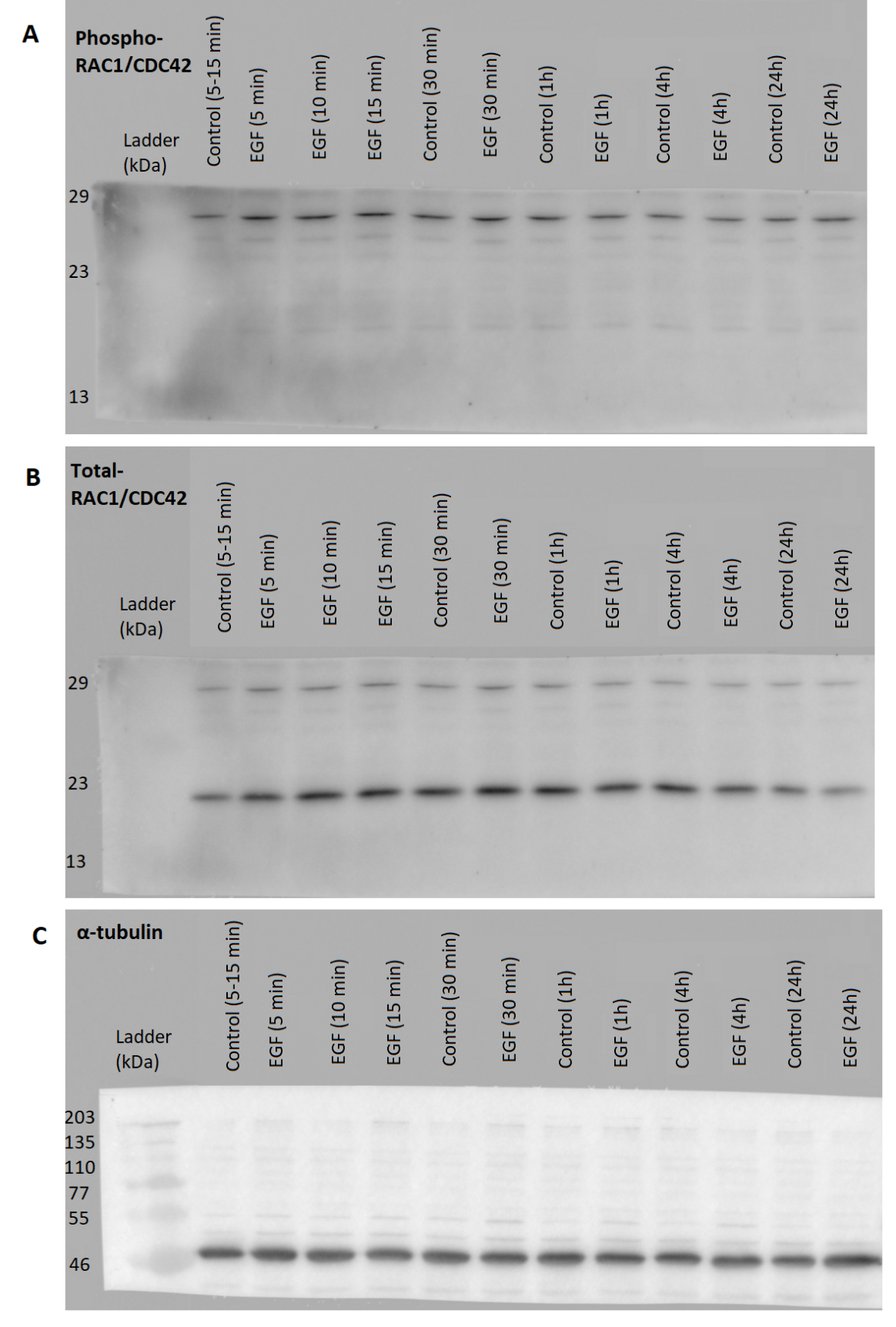
**

**Supplementary Figure 4. Full western blot images with the corresponding ladder showing RAC1/CDC42 expression.** Western blot images of A549 cells stimulated with EGF showing expression of **(A)** Phospho-RAC1/CDC42 (28 kDa) and **(B)** Total-RAC1/CDC42 protein (21 kDa) in a time-dependent manner. **(C)** α-tubulin (56 kDa) served as an internal control for protein loading. For each time point, the corresponding controls (i.e., cells without EGF treatment) are shown next to the EGF treated samples. After the transfer, the membranes were cut in order to perform the immunoblotting for different proteins of interest in the same membrane. This can be visualized by the corresponding ladder (mPAGE® Color Protein Standard, Cat. #MPSTD4, Sigma-Aldrich) according to the band size (kDa).

*
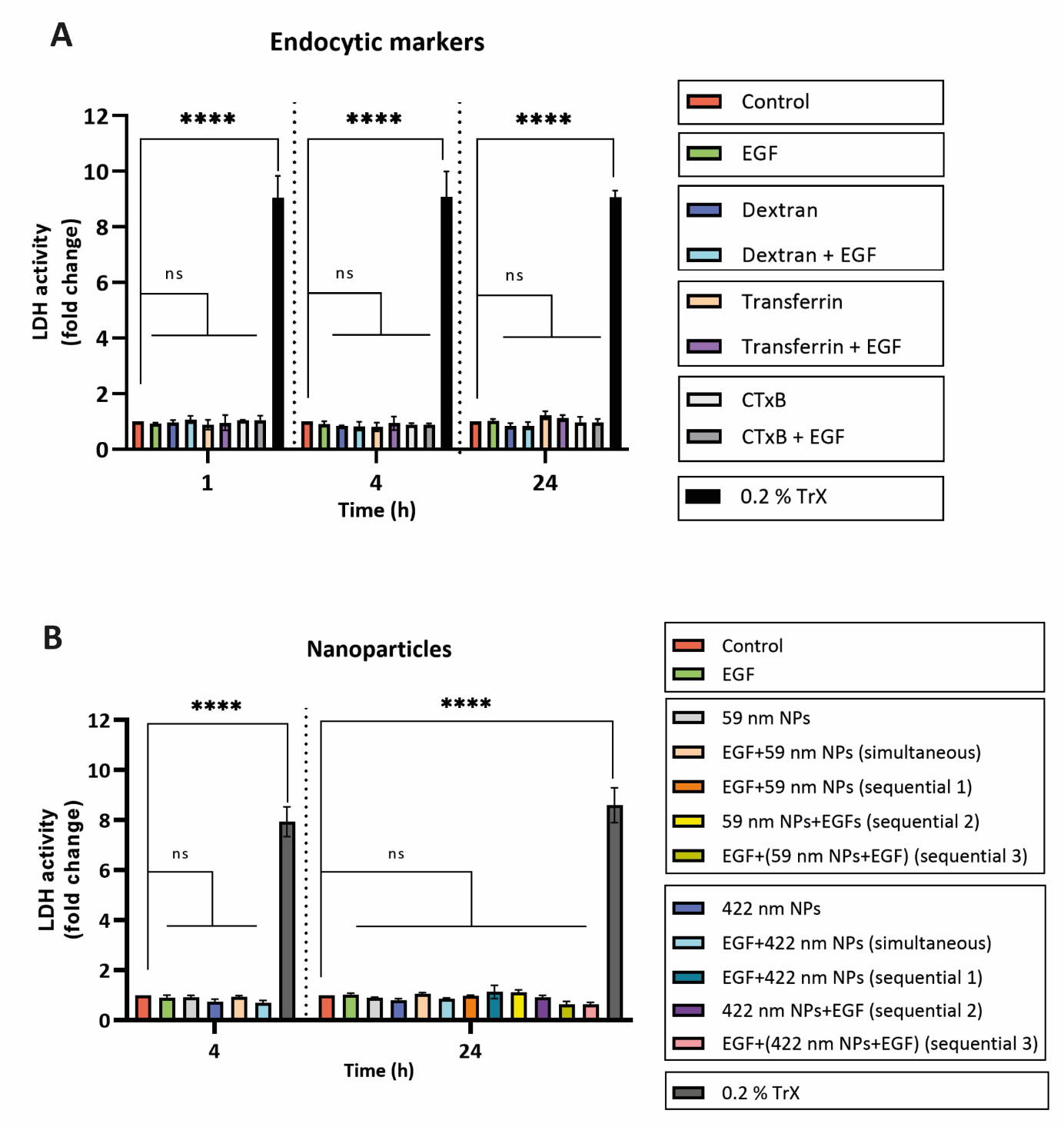
*

**Supplementary Figure 5. Cytotoxicity of endocytic markers and SiO_2_-BDP FL NP in the absence and presence of EGF, assessed via lactate dehydrogenase (LDH) assay.** (A) A549 cells were exposed to dextran (250 µg/mL), transferrin (10 µg/m) and cholera toxin subunit B (CTxB; 100 ng/mL alone or their combination with EGF (100 ng/mL) for 1 h, 4 h, and 24 h. (B) Cell viability upon exposure to 59 nm SiO_2_ NP and 422 nm SiO_2_ NP (both 50 µg/mL) in the absence or presence of EGF (100 ng/mL). Different co-exposure scenarios are shown. Data were normalized to the negative control and expressed as a fold change over the negative control. 0.2 % TritonX served as a positive control for membrane rupture. No cytotoxic effect was observed for all treatments. Data from each experiment were obtained from three biological replicates and are presented as mean ± standard deviation. Statistical significance was determined by One-way ANOVA (GraphPad Prism): **** p < 0.0001, ns - not significant.

*
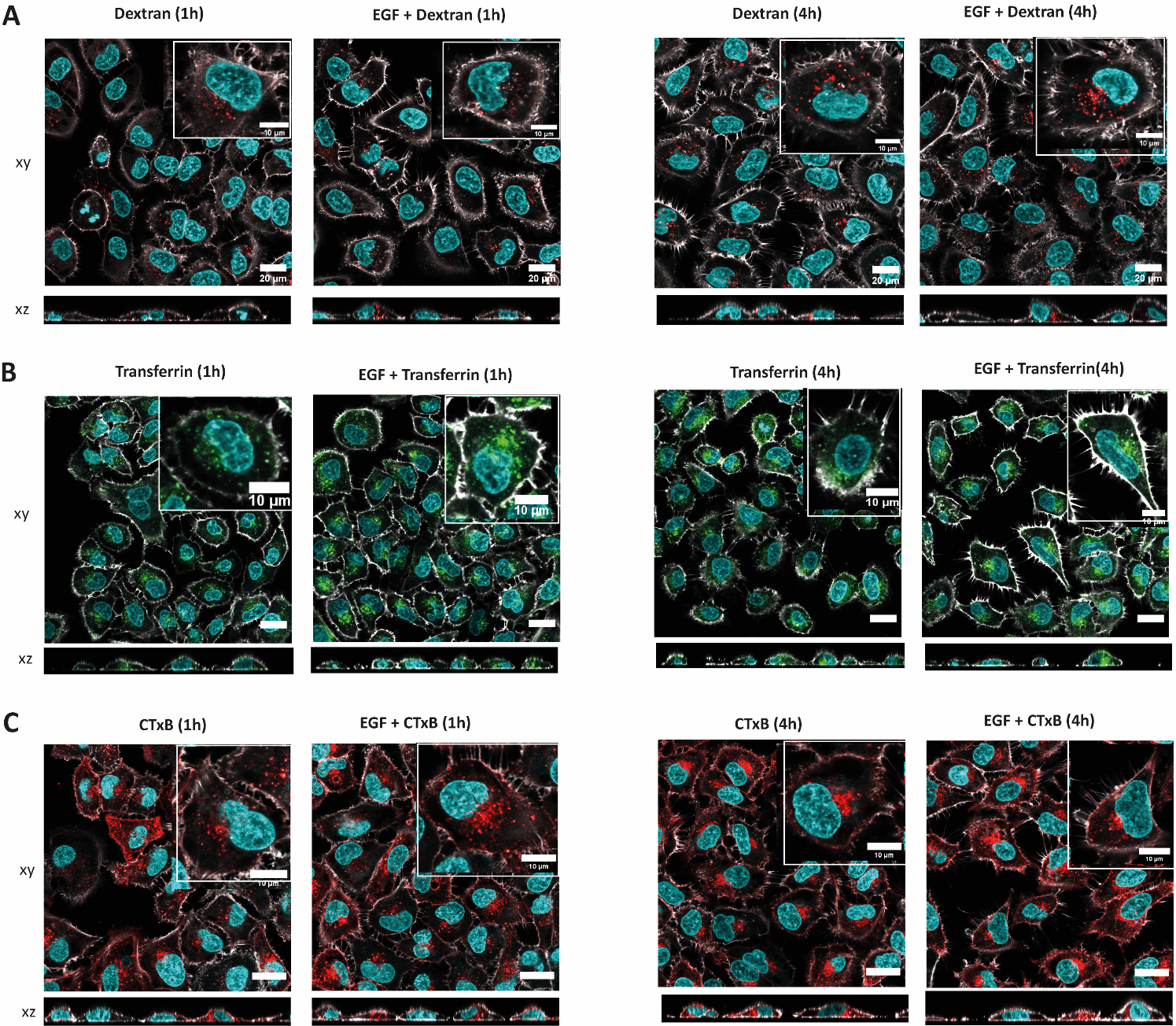
*

**Supplementary Figure 6. Cellular uptake of endocytic markers at 1h and 4h time-point.** Confocal laser scanning micrographs showing the uptake (**A**) 70 kDa dextran (macropinocytosis marker; red) and (**B**) transferrin (clathrin-mediated endocytosis marker; green) and (C) cholera toxin subunit B (CTxB; caveolin-mediated endocytosis marker; red) in the absence and presence of 100 ng/mL EGF after 1 h and 4 h of exposure. Grey: F-actin, Cyan: nuclei. Scale bar: 20 µm. Images of single cells are shown in the top right corner, scale bar: 10 µm.


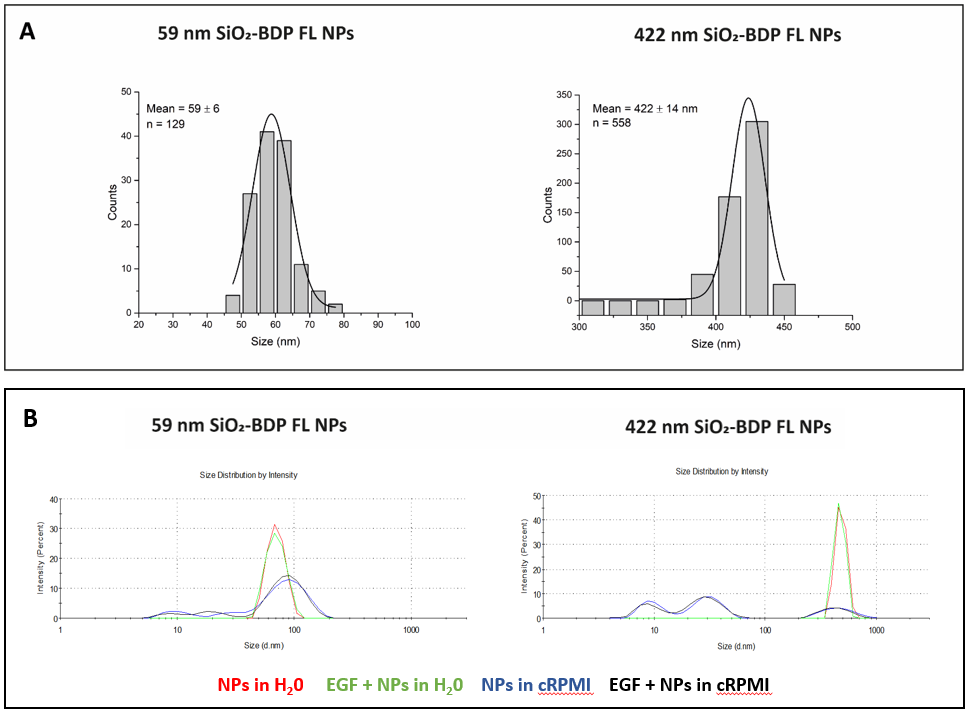


**Supplementary Figure 7. SiO_2_-BDP FL NPs characterization.** (**A**) Histograms showing the size distributions of 59 nm and 422 nm SiO_2_-BDP FL NPs assessed from transmission electron micrographs using Fiji software. Left figure is reprinted with permission from Susnik *et al*., Cells, 2020, 9(9). Right figure reprinted with permission from A. Lee, *et al.,* Nanoscale, 2022, 14, 15141 (CC BY-NC 3.0). (**B**) Hydrodynamic size distribution of 59 nm and 422 nm SiO_2_-BDP FL NPs measured via dynamic light scattering (DLS) (Malvern Zetasizer Nano ZS at 37°C, scattering angle 173° and laser wavelength 633 nm). Hydrodynamic diameters of 59 nm and 422 nm SiO_2_-BDP FL NPs in Milli-Q water and cRPMI have been assessed in the absence and presence of EGF and show no differences. A single peak was observed when NPs were dispersed in Milli-Q water, indicating stable NPs dispersion. In cRPMI, three peaks were observed corresponding to proteins and NPs.


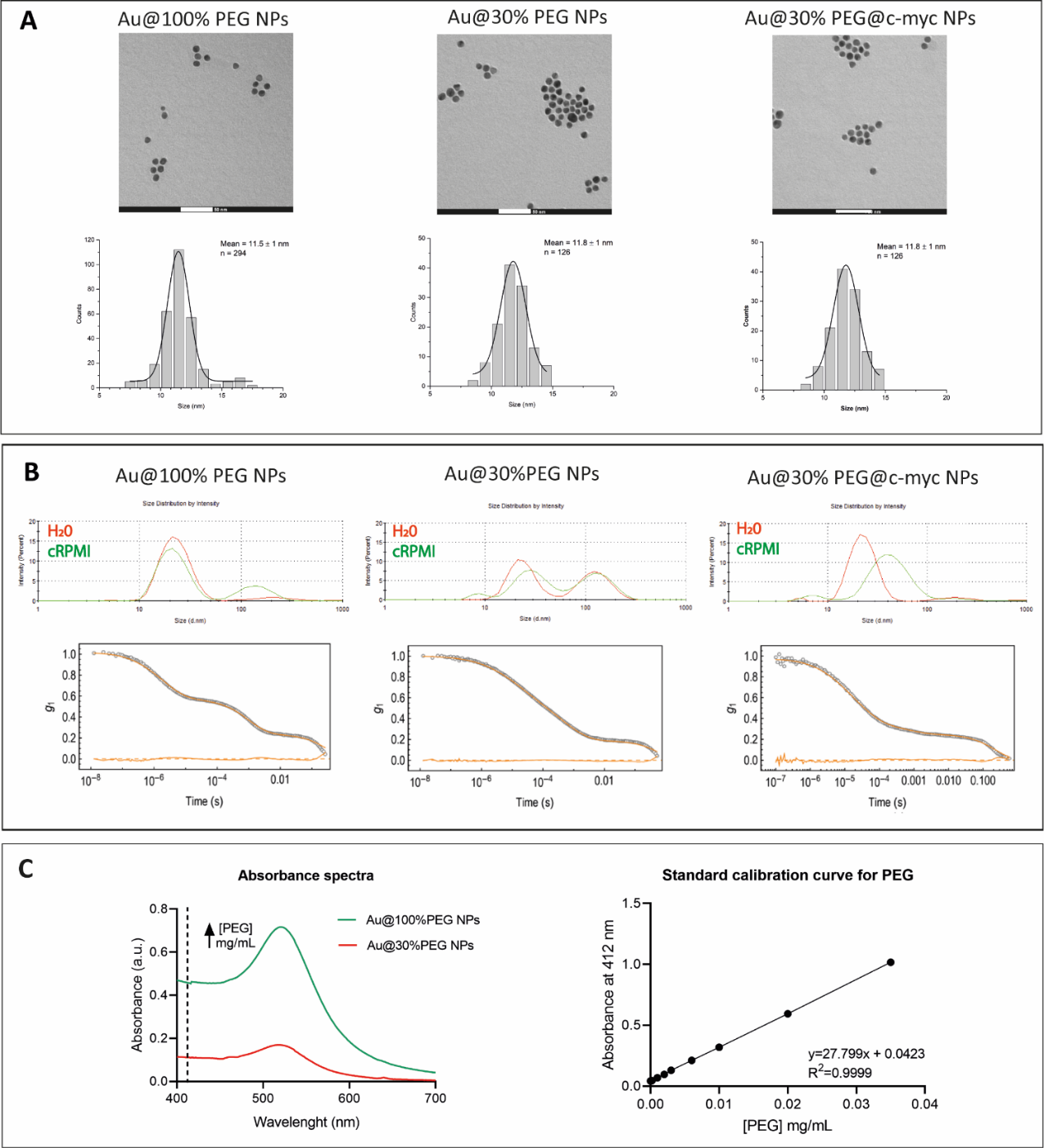


**Supplementary Figure 8. Au@PEG NPs characterization.** (**A**) Representative transmission electron micrographs (TEM) of Au@30%PEG NPs, Au@30%PEG NPs, and Au@30%PEG@c-myc NP and the corresponding histograms showing the size NPs distributions determined from transmission electron micrographs. (**B**) Hydrodynamic size distribution of Au@100%PEG NPs, Au@30%PEG NPs, and Au@30%PEG@c-myc NPs measured via dynamic light scattering (DLS) in water and cRPMI (Malvern Zetasizer Nano ZS at 37°C, scattering angle 173° and laser wavelength 633 nm). The first peak indicates single colloidal NPs dispersion. We hypothesize that the differences in NPs size distribution in Milli-Q water and cRPMI can be either due to the presence of proteins in cRPMI that can influence the NP’s stability when adsorbed on the surface (protein corona). To complement DLS data measured by Malvern, the auto-correlation functions for each NPs dispersed in cRPMI have been analyzed with a DLS spectrometer LS Instruments AG (Fribourg, Switzerland) at the scattering angle of 90° and laser wavelength 660 nm. Data is represented as fit (+ residuals) corresponding to the estimated distribution of the intensity-weighted hydrodynamic radius. As it can be appreciated from the auto-correlation function, we noted heterogeneity among NPs colloidal distribution in cRPMI. **(C)** Absorbance spectra of Au@30%PEG NPs and Au@100%PEG NPs used to determine the surface coverage with PEG and the standard calibration curve with increasing concentrations of PEG. Using the equation derived from the standard curve and the measured absorbance value of the NPs at 412 nm, we can determine the extent of NPs coverage with PEG. Specifically, a concentration of 0.01 mg/mL corresponds to 100% PEG coverage, while a concentration of 0.003 mg/mL corresponds to 30% PEG coverage.


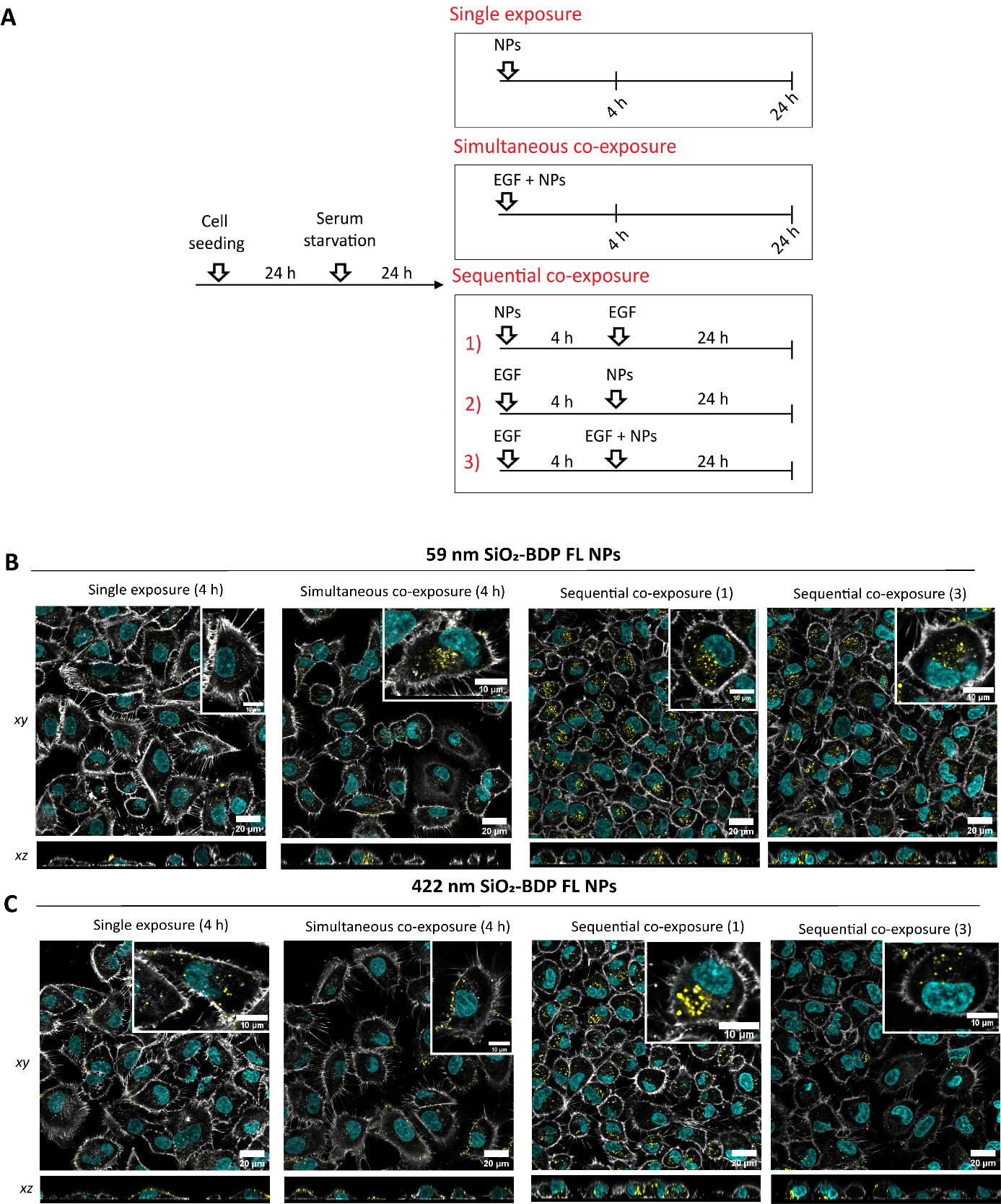


**Supplementary Figure 9. Uptake of 59 nm and 422 nm SiO_2_-BDP FL NPs upon cell stimulation with EGF. (A)** Schematic representation of different experimental co-exposure scenarios. Confocal laser scanning microscopy data showing the association of (**B**) 59 nm SiO_2_-BDP FL NP and (**C**) 422 nm SiO_2_-BDP FL NP with A549 cells under different experimental set-ups: Single exposure (4 h), simultaneous co-exposure with EGF and sequential co-exposure with EGF (scenarios 1 and 3). Yellow: NPs, Grey: F-actin, Cyan: nuclei. Scale bar: 20 µm. Zoom-in images of single cells are shown in insets (scale bar: 10 µm).


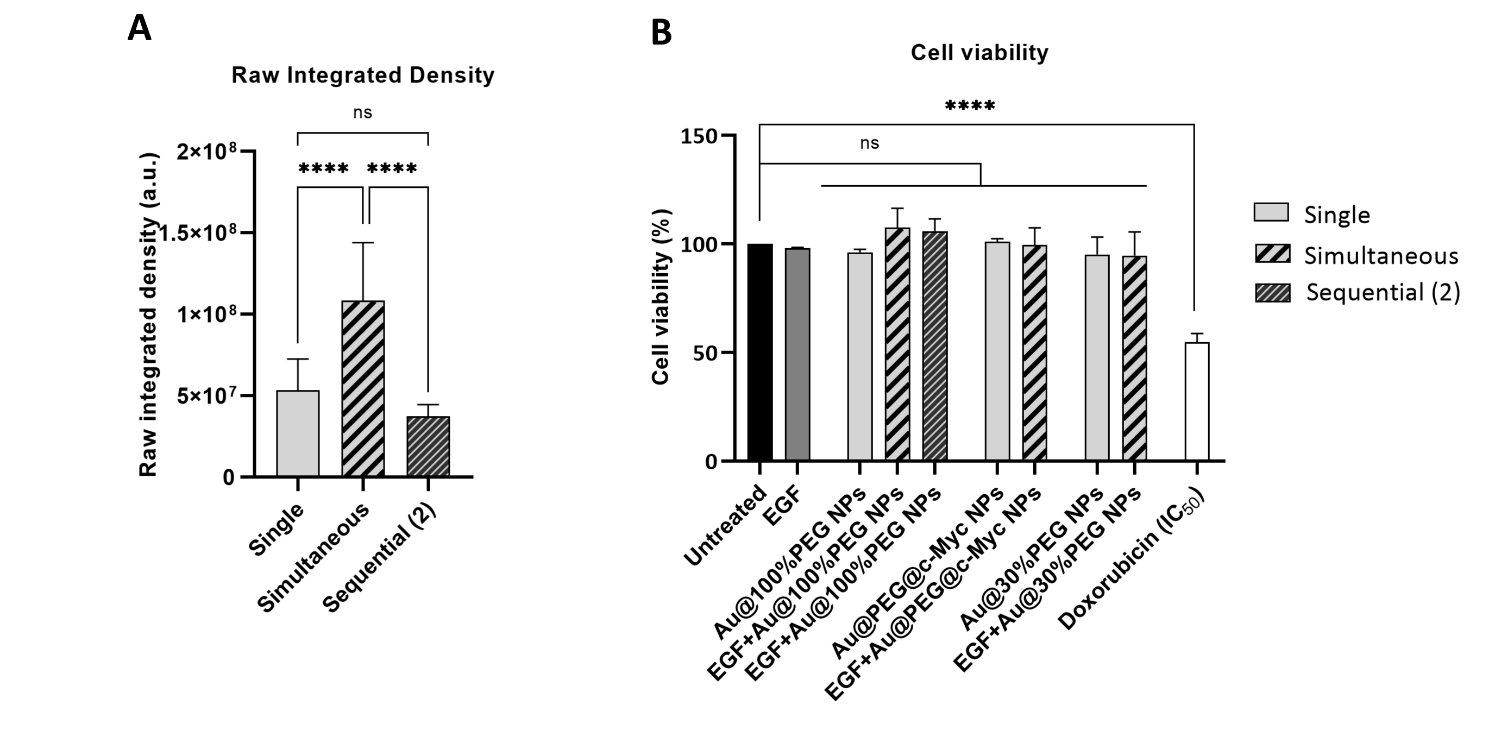


**Supplementary Figure 10. Raw integrated density and cell viability of different PEGylated Au NPs.** (**A**) Comparison of the raw integrated densities of Au@100%PEG NPs under three different co-exposure conditions. Data for each condition were obtained from 10 individual cells from darkfield cell imaging. Statistical significance was determined by One-way ANOVA (GraphPad Prism): **** p < 0.0001, ns - not significant. (**B**) MTS assay of cell viability/proliferation indicated by mitochondrial activity after A549 cells treatment with Au@PEG NPs in the absence or presence of EGF. Untreated cells and cells treated with doxorubicin in concentration 15 μM (IC50) for 24 h served as controls for metabolic activity. The mitochondrial activity in untreated cells was assigned a value of 100 %. Experiments were performed in triplicates and are presented as mean ± standard deviation.

**Supplementary Table 1.** Maintenance conditions for different cell lines.

| **Cell line** | **Provider** | **Cell culture medium** | **Supplements** | **Subcultivation** |
| --- | --- | --- | --- | --- |
| **A549-EGFR-GFP** | Sigma-Aldrich, CLL1141 | RPMI-1640 (Cat. 42401-018, Gibco) | 10 % hi-FBS  2 mM L-Glu  100 µg/mL P/S | Detach with 0.05 % Trypsin/EDTA, centrifuge 5 min at 300 x g, and subcultivate every 3-4 days (dilution factor 1:8). |
| **ARPE-19** | ATCC®  CRL-2302 | DMEM-F12 (Cat. 11330-032, Gibco) | 10 % hi-FBS  10,000 Units/mL P/S  50 mM 2-mercaptoethanol  250 µg/mL amphotericin B  100 mM sodium pyruvate  100 g/L D-glucose  100x NEAA | Detach with 0.05 % Trypsin/EDTA, centrifuge 5 min at 300 x g, subcultivate every 2-3 days (dilution factor 1:3 or 1:5). |
| **HeLa** | ATCC®  CCL-17™ | RPMI-1640 (Cat. 42401-018, Gibco) | 10 % hi-FBS  2 mM L-Glu  100 µg/mL P/S | Detach with 0.05 % Trypsin/EDTA, centrifuge 5 min at 300 x g, subcultivate every 3-4 days (dilution factor 1:8). |
| **Calu-3** | ATCC®  HTB-55 | MEM-Glutamax (Cat. 41090-093, Gibco) | 10% hi-FBS  1% NEAA  100 µg/mL P/S  2% amphotericin B | Detach with 0.05 % Trypsin/EDTA, centrifuge 5 min at 300 x g, subcultivate once per week (seeding density 2x10^6^ cells/10 mL of medium). |
| **Caco-2** | DSMZ, ACC169 | MEM (Cat. 21090022, Gibco) | 20 % hi-FBS  2 mM L-Glu  100 µg/mL P/S | Detach with 0.05 % Trypsin/EDTA, centrifuge 5 min at 300 x g, subcultivate every 2 days (seeding density 7.5x10^5^ cells/10 mL of medium). |
| **HT-29** | ATCC®  HTB-38™ | DMEM (Cat. 41965039, Gibco) | 10% hi-FBS  2 mM L-Glu  1% NEAA | Detach with 0.05 % Trypsin/EDTA, centrifuge 5 min at 300 x g, subcultivate every 2 days (seeding density 3x10^6^ cells/10 mL of medium). |
| **THP-1** | ATCC®  TIB-202™ | RPMI-1640 (Cat. 42401-018, Gibco) | 10 % hi-FBS  0.25 g/ml D-glucose  100 mM sodium pyruvate  2 mM L-Glu  100 µg/mL P/S  50 mM 2-mercaptoethanol | Centrifuge cells in suspension 5 min at 200 x g, transfer 2×10^6^ cells into a new T25 flask containing 10 mL medium with 2-mercaptoethanol. To differentiate monocytes into macrophages add 100 nM PMA for 2 days. Detach differentiated cells with 1x accutase. |

Abbreviations: ATCC - American Type Culture Collection, DMEM - Dulbecco's Modified Eagle's cell culture medium, EDTA - Ethylenediaminetetraacetic acid, hi-FBS - Heat-inactivated fetal bovine serum, L-Glu – L-Glutamine, NEAA - non-essential amino acids, nhi-FBS - Non-heat inactivated FBS, P/S – penicillin/streptomycin, PMA – phorbol 12-myristate-13-acetate, RPMI - Roswell Park Memorial Institute cell culture medium.

**Supplementary Table 2.** Primer sequences used in RT-qPCR. *FW:* Forward primer. *RV:* Reverse primer.

| **Gene symbol** | **Gene name** | **Sequence (5'->3')** | **Product length (bp)** |
| --- | --- | --- | --- |
| ***RAC1*** | Rac family small GTPase 1 | FW: TGGCTAAGGAGATTGGTGCTG  RV: CGGATCGCTTCGTCAAACAC | 88 |
| ***GAPDH*** | Glyceraldehyde-3-phosphate dehydrogenase | FW: GTCGGAGTCAACGGATTTGG  RV: GCCATGGGTGGAATCATATTGG | 147 |
| ***YWHAZ*** | Tyrosine 3-monooxygenase/tryptophan 5-monooxygenase activation protein zeta | FW: GCTGGTGATGACAAGAAAGGGAT  RV: GTTAAGGGCCAGACCCAGTC | 120 |
| ***c-MYC*** | Cellular myelocytomatosis oncogene | FW: GCTCATTTCTGAAGAGGACTTGT  RV: GGCAGTTTACATTATGGCTAAATC | 229 |
| ***18S*** | 18S ribosomal RNA | FW: GTAACCCGTTGAACCCCATT  RV: CCATCCAATCGGTAGTAGCG | 151 |

**Supplementary Table 3.** RT-qPCR cycling conditions to amplify *RAC1, GAPDH* and *YWHAZ* genes using 7500 fast real-time PCR system (Applied Biosystems) and *c-MYC* and *18S* genes using Qiagen Rotor-Gene Q.

| **Cycles** | **Temperature** | **Time** | **Main reaction** |
| --- | --- | --- | --- |
| ***RAC1, GAPDH* and *YWHAZ* genes** | | | |
| 1 | 60°C | 20 min | reverse transcription |
| 1 | 95°C | 3 min | polymerase activation / denaturation |
| 40 | 60°C | 30 sec | annealing / extension |
| ***c-MYC* and *18S* genes** | | | |
| 1 | 50°C | 20 min | reverse transcription |
| 1 | 95°C | 5 min | polymerase activation |
| 35 | 95°C | 15 sec | denaturation |
|  | 60°C | 15 sec | annealing / extension |
